# The pathogenic role of c-Kit+ mast cells in the spinal motor neuron-vascular niche in ALS

**DOI:** 10.1186/s40478-021-01241-3

**Published:** 2021-08-13

**Authors:** Mariángeles Kovacs, Catalina Alamón, Cecilia Maciel, Valentina Varela, Sofía Ibarburu, Lucas Tarragó, Peter H. King, Ying Si, Yuri Kwon, Olivier Hermine, Luis Barbeito, Emiliano Trias

**Affiliations:** 1 Institut Pasteur de Montevideo, 2020 Montevideo, Uruguay; 2 Department of Neurology, University of Alabama, Birmingham, AL 35294 USA; 3 Birmingham Veterans Affairs Medical Center, Birmingham, AL 35295 USA; 4 Imagine Institute, Hôpital Necker, Paris, France; 5 INSERM UMR 1163, Laboratory of Cellular and Molecular Mechanisms of Hematological Disorders and Therapeutic Implications, Paris, France; 6 Imagine Institute, Paris Descartes–Sorbonne Paris Cité University, Paris, France; 7 CNRS ERL 8254, Paris, France; 8 Laboratory of Excellence GR-Ex, Paris, France; 9 Equipe Labélisée par la Ligue Nationale contre le cancer; AB Science; Department of Hematology, Necker Hospital, Paris, France; 10 Centre national de référence des mastocytoses (CEREMAST), Paris, France

**Keywords:** Mast cells, Motor neuron-vascular niche, ALS, Masitinib, Spinal cord

## Abstract

Degeneration of motor neurons, glial cell reactivity, and vascular alterations in the CNS are important neuropathological features of amyotrophic lateral sclerosis (ALS). Immune cells trafficking from the blood also infiltrate the affected CNS parenchyma and contribute to neuroinflammation. Mast cells (MCs) are hematopoietic-derived immune cells whose precursors differentiate upon migration into tissues. Upon activation, MCs undergo degranulation with the ability to increase vascular permeability, orchestrate neuroinflammation and modulate the neuroimmune response. However, the prevalence, pathological significance, and pharmacology of MCs in the CNS of ALS patients remain largely unknown. In autopsy ALS spinal cords, we identified for the first time that MCs express c-Kit together with chymase, tryptase, and Cox-2 and display granular or degranulating morphology, as compared with scarce MCs in control cords. In ALS, MCs were mainly found in the niche between spinal motor neuron somas and nearby microvascular elements, and they displayed remarkable pathological abnormalities. Similarly, MCs accumulated in the motor neuron-vascular niche of ALS murine models, in the vicinity of astrocytes and motor neurons expressing the c-Kit ligand stem cell factor (SCF), suggesting an SCF/c-Kit-dependent mechanism of MC differentiation from precursors. Mechanistically, we provide evidence that fully differentiated MCs in cell cultures can be generated from the murine ALS spinal cord tissue, further supporting the presence of c-Kit+ MC precursors. Moreover, intravenous administration of bone marrow-derived c-Kit+ MC precursors infiltrated the spinal cord in ALS mice but not in controls, consistent with aberrant trafficking through a defective microvasculature. Pharmacological inhibition of c-Kit with masitinib in ALS mice reduced the MC number and the influx of MC precursors from the periphery. Our results suggest a previously unknown pathogenic mechanism triggered by MCs in the ALS motor neuron-vascular niche that might be targeted pharmacologically.

**Supplementary Information:**

The online version contains supplementary material available at 10.1186/s40478-021-01241-3.

## Introduction

The pathogenesis of amyotrophic lateral sclerosis (ALS) is multifactorial and remains partially understood. However, recent evidence suggests that peripheral immune cells such as lymphocytes, monocytes/macrophages, and mast cells (MCs), significantly contribute to deleterious inflammation and disease progression [3, 5, 6, 49]. Evidence indicate that peripheral MCs play a pathogenic role in ALS, through close contact with motor axons and NMJs (neuromuscular junctions) undergoing active peripheral motor pathway degeneration in ALS [48, 49]. While MCs have been described in the CNS of ALS subjects [19], little is known, however, about the mechanisms controlling the MC influx to the Central Nervous System (CNS) and their interaction with motor neurons and perineuronal microvasculature in the spinal cord.

MCs are effectors cells of the innate immune system derived from hematopoietic myeloid precursors that can be found in low numbers in all vascularized tissues including specific brain regions [14, 26, 52]. MC precursors expressing the tyrosine kinase receptor c-Kit, leave the bone marrow to enter the circulation and are recruited to tissues through a trans-endothelial passage, and are often found close to blood vessels [26]. Immature MCs achieve maturation within the local tissue microenvironment, a process that involves changes in phenotype and accumulation of granules containing neuroactive and vasoactive molecules, proteases, and proteoglycans [10, 31, 56]. Functionally, MCs play a key role in inflammatory processes via controlled degranulation (secretion) of several cytokines, trophic factors, proteases, lipid metabolites, and nitric oxides [42]. MC accumulation and activation in the CNS pathologies such as multiple sclerosis are known to mediate chronic neuroinflammation, microglial activation, and disruption of the blood–brain barrier (BBB) and brain-spinal cord barrier (BSCB) [12, 40, 43]. Although a growing body of evidence supports the pathogenic role of MCs in neurodegenerative conditions, little is known about the potential role of MCs inducing motor neuron damage and/or vascular dysfunction in ALS.

The tyrosine kinase receptor c-Kit (KIT or CD117) belongs to a family of transmembrane growth factor receptors [39]. The c-Kit specific ligand is Stem Cell Factor (SCF), also known as mast cell growth factor [13]. While c-Kit is expressed extensively in hematopoietic cells, it is generally lost during the differentiation process, except for MCs that retain c-Kit through their lifespan [27]. Thus, the SCF/c-Kit pathway is required for the survival, differentiation, and degranulation of MCs [27]. Pharmacological inhibition of c-Kit in ALS murine models with masitinib results in significant amelioration of paralysis progression and a sharp decrease in MC infiltration into the degenerating peripheral motor pathways and NMJs [47–50]. Masitinib is a tyrosine kinase inhibitor drug that also targets type III kinases [11], including CSF-1R in microglia, monocytes, and macrophages, resulting in decreased microgliosis in the spinal cord [47–49]. Evidence from clinical trials indicate that masitinib exerts therapeutic effects in ALS [24, 32], as well as in progressive forms of multiple sclerosis [53] and Alzheimer’s disease [38], indicating therefore that it can target neuroinflammatory and neurodegeneration processes through multifaceted mechanisms, including MCs downregulation.

Because MCs are mainly localized nearby blood vessels [4] and cluster around degenerating motor nerve terminals in ALS [49], we analyzed whether MCs also infiltrated around the motor neuron cell bodies and surrounding microvasculature elements in the spinal cord of both ALS subjects and murine models. We report for the first time that MCs accumulate in significant numbers within the motor neuron-vascular niche in ALS and provide evidence for undifferentiated MCs trafficking from the periphery through microvasculature that shows remarkable morphological abnormalities. Moreover, we show in ALS mice that MCs number and trafficking into the spinal cord were downregulated by masitinib, a drug that modulates MCs activity via inhibition of c-Kit, LYN, and FYN kinases, with subsequent anti-inflammatory and neuroprotective effects in ALS [32, 47].

## Material and methods

### Human tissue collection

The collection of post-mortem human ALS and control samples was approved by The University of Alabama at Birmingham (UAB) Institutional Review Board (Approved IRB Protocol: X091222037 to Dr. Peter H. King). All ALS patients were cared for at UAB and detailed clinical records were available. Control samples were age-matched and were harvested from patients who expired from non-neurological causes. None of the ALS cases correspond to familiar cases associated with SOD1 mutations. The average collection time after death was less than 10 h (Additional file 1: Table 1). All tissues were collected by Peter H. King and Ying Si.

### Human spinal cord immunohistochemistry

In this study, 10 μm spinal cord paraffin sections were sliced using a microtome. Following deparaffinization, antigen retrieval was performed in 10 mM citrate buffer pH 6 using a steamer cooker, reaching 95 °C for 30 min. Then, slices were cooled down in the same citrate buffer at room temperature for 30 min and washed with PBS for 2 h. After washing, slices were blocked and permeabilized in BSA 5%/Triton X-100 0.5% for 2 h at room temperature. Primary antibodies were incubated in BSA 1%/Triton X-100 0.5% at 4 °C overnight. After washing, fluorophore-conjugated secondary antibodies were incubated for 3 h at room temperature. After PBS washing, Mowiol medium (Sigma, St. Louis, MO, USA) was used for mounting. Only ventral lumbar spinal cord sections were analyzed. Motor neuron somas were identified in the ventral spinal cord by typical morphology and nuclei. Saturated DAPI staining was used to better differentiate motor neuron somas as previously described [28]. For diaminobenzidine (DAB) staining, biotinylated anti-rabbit and anti-mouse secondary antibodies were used after primary incubation. The protocol described in the VECTASTAIN Elite ABC-HRP Kit (Vector Laboratories, #PK-6101) was followed together with the ImmPACT DAB substrate (SK-4105). After washing, the hematoxylin staining protocol was performed for 3 min to stain nuclei.

### Animals

Mice used in this study were obtained from The Jackson Laboratory, Bar Harbor, MA, USA. Animals were housed in a centralized animal facility with a 12-h light–dark cycle with ad libitum access to food and water. Male mice B6SJL-Tg(SOD1*G93A)1Gur/J mice, over-expressing human SOD1 mutation (G93A SOD1) were used for further breeding to maintain the line. Perfusion with fixative was performed under 90% ketamine/10% xylazine anesthesia and all efforts were made to minimize animal suffering, discomfort, or stress. All mice were weighed and evaluated for motor activity daily. Disease onset was determined for each animal when pronounced muscle atrophy was accompanied by an abnormal gait, typically expressed as subtle limping or dragging of one hind limb (~ 120 days old). Also, male SOD1^G93A^ rat progeny, purchased from Taconic bioscience (NTac:SD-Tg (SOD1G93A)L26H), were used for further breeding to maintain the line [11]. Rats were housed in a centralized animal facility with a 12-h light–dark cycle with ad libitum access to food and water. Perfusion with fixative was performed under 90% ketamine/10% xylazine anesthesia and all efforts were made to minimize animal suffering, discomfort, or stress. All procedures using laboratory animals were performed following the national and international ARRIVE guidelines and were approved by the Institutional Animal Committee for animal experimentation. This study was carried out in strict accordance with the Institut Pasteur de Montevideo ethical committee’s requirements (CEUA Approved protocols: #005-17, #016-19, #005-20, #007-20, to Dr. Luis Barbeito) and the national law (Number 18.611) for animal experimentation that follows the Guide for the Care and Use of Laboratory Animals of the National Institutes of Health (USA).

### Immunohistochemistry of mice spinal cord

At least four animals of each condition were used for the immunohistochemistry experiment. Non-transgenic (Non-Tg) mice of 150–160 days and transgenic SOD1^G93A^ symptomatic mice of 120–130 days (SOD1^G93A^ Onset) and 150–160 days (SOD1^G93A^ Symp) were used to perform experiments. Animals were deeply anesthetized and transcardial perfusion was performed with paraformaldehyde 4% (v/v) in PBS pH = 7.4. Fixed spinal cords were cryopreserved in 30% sucrose (Sigma, St. Louis, MO, USA) at 4 °C. After 72 h, tissue was embedded in Tissue-Tek (Sakura), sectioned (transversal) using a cryostat, and collected on gelatin-coated slides. Then, 25 μm sections were blocked for 2 h at room temperature in 5% BSA, 0.3% Triton X-100/2% Goat Serum in PBS, incubated with primary antibodies overnight at 4 °C in 1% BSA/0.3% Triton X-100/0.4% Goat Serum. After washing, fluorophore-conjugated secondary antibodies were incubated for 2 h at room temperature in 1% BSA/0.3% Triton X-100/0.4% Goat Serum. To determine primary antibodies’ specificity, immunohistochemistry was carried out in the absence of primary antibodies. Non-significant immunofluorescence was detected with secondary antibodies incubation. DPX mounting medium (Sigma, St. Louis, MO, USA) was used for mounting. DAB staining was performed as described above.

### Histochemistry quantitative analysis

MCs in the ventral horn of the lumbar spinal cord sections were identified by the typical granular morphology and tryptase+/chymase+, c-kit+/chymase+, c-Kit+/Cox-2+, chymase+/Cox-2+or c-Kit+/CD45+ immunohistochemical staining as well as c-Kit+/CFSE+, c-Kit+ or CFSE+ infiltrating MC precursors, using a stereological approach. The counting was performed only in the area surrounding motor neuron cell bodies within a radius of 150 μm of the soma. The counting was carried out using maximum-intensity projection confocal microphotographs with a magnification of 63x. At least fifteen sections per spinal cord were analyzed (*n* = 4 for mice spinal cord analysis, *n* = 5 for human spinal cord analysis). Image J software was used for analysis.

For co-expression of SCF with the astrocyte marker, GFAP was carried out using Image J software as previously described [28]. At least fifteen sections per spinal cord per animal per condition (*n* = 4) were used. The number of c-Kit+ cells surrounding SCF+ astrocytes and SCF+ motor neurons was assessed using ImageJ software, counting the positive cells in maximum-intensity projection confocal microphotographs with a magnification of 63x. At least fifteen sections per spinal cord per condition were analyzed (*n* = 4 for mice spinal cord analysis).

### Toluidine blue staining of mast cells.

For the MC analysis based on metachromasia observation, as previously described [48], 10 μm sections of human (paraffin sections) and mice (fixed frozen sections) spinal cord were microtome and cryostat sliced and mounted in gelatin-coated slides respectively. Also, cultured cells obtained from the SOD1^G93A^ rat spinal cord and mounted in slides were used. Slides were washed and hydrated 2 times in distilled water for 10 min and embedded in 1% toluidine blue solution for 10–20 min for human and mice tissue and 5 min in case of cultured cells. Then, slides were washed in distilled water for 5 min and dehydrated for 3 min in 70% ethanol, 3 min in 95% ethanol, and finally 2 min in 100% ethanol. Slides were cleared in xylene twice, 3 min each, and finally mounted in DPX (Sigma-Aldrich). Images (at × 100 magnification) were acquired using an Olympus CX41 microscope connected to an Evolution LC Color camera and using ImagePro Express software for acquisition.

### Flow cytometry analysis of the mice spinal cord

Spinal cords from both, Non-Tg or SOD1^G93A^ symptomatic mice were dissected, and the meninges were carefully removed in animals deeply anesthetized as described above. Spinal cords were mechanically chopped and enzymatically dissociated using 0.25% trypsin for 5 min at 37 °C. Fetal Bovine Serum (FBS) 10% (vol/vol) in PBS was then added to halt trypsin digestion. Repetitive pipetting thoroughly disaggregated the tissue, which was then collected on an 80 μm mesh strainer and spun down. The resulting filtered cell suspensions were re-suspended in PBS-FBS 2%-1 mM EDTA.

Flow cytometry analysis was performed as previously described [18]. Briefly, single-cell suspensions from the spinal cord were incubated with fluorochrome-conjugated antibodies against the following antigens: CD45-PerCP (BioLegend, #103130) as an infiltrating hematopoietic cell marker, and c-Kit conjugated to biotin (Abcam, #ab25022) in PBS-FBS 2%-1 mM EDTA for 20 min at 4 °C. After incubation and washing, single-cell suspensions were incubated with 1:500 Streptavidin- Alexa Fluor 405 (Thermo Fisher Scientific, #S21375). Analysis was performed on an Attune NxT Flow Cytometer (Thermo Fisher Scientific, Waltham, MA, USA). Post-acquisition analysis was assessed using FlowJo software.

### Cultures of mast cell precursors from the symptomatic SOD1^G93A^ rats’ spinal cord

Primary cell cultures were obtained from the spinal cord of symptomatic SOD1^G93A^ rats according to the procedures described by Trias et al., 2013 with modifications [46]. Briefly, animals were euthanized by overdosing with ketamine/xylazine, and the spinal cords were carefully dissected on ice with meninges removal. Then, spinal cords were chopped finely and dissociated with 0.25% trypsin in a calcium-free buffer for 5 min at 37 °C. Trypsin treatment was stopped by adding RPMI 1640/15% (vol/vol) FBS. The resulting extract was passed through an 80-μm mesh to eliminate tissue debris and then was spun. The pellet was resuspended in a mast cell culture medium [RPMI 1640/15% (vol/vol) FBS, sodium pyruvate (1 mM), β-mercaptoethanol (50 μM)] and then placed in 75-cm^2^ tissue culture flasks in presence of SCF and IL-3 (20 ng/mL) freshly added every 48 h. Non-adherent cells were resuspended with the culture medium subsequently replaced and detached cells were discarded every 48 h. Cells were analyzed by flow cytometry at 2 and 7 days and characterized by immunocytochemistry at 14 days, as described below.

### Flow cytometry analysis of cultured c-Kit+ mast cells precursors from SOD1^G93A^ spinal cord

After 2 and 7 days in vitro (DIV), the c-Kit+ population was analyzed by flow cytometry. Removal of cell debris was performed using the Debris Removal Solution (Miltenyi Biotec, #130-109-398) according to the manufacturer´s instructions. Briefly, the cell suspension was resuspended in cold PBS 1X, and 900 μL of Debris Removal Solution were added and overlaid gently with 4 mL of cold PBS. The cell suspension was centrifuged at 4 °C and 3000xg for 10 min. Two top phases were completely aspirated and discarded. Cold PBS was added to a final volume of 15 mL, and the cell suspension was centrifuged at 4 °C and 1000xg for 10 min. Finally, the supernatant was completely aspirated, and cells were resuspended carefully in PBS-FBS 2%-1 mM EDTA. Then, single-cell suspensions were incubated with c-Kit conjugated to biotin in PBS-FBS 2%-1 mM EDTA for 20 min at 4 °C. After incubation and washing, single-cell suspensions were incubated with 1:500 Streptavidin- Alexa Fluor 405. Analysis was performed on an Attune NxT Flow Cytometer (Thermo Fisher Scientific, Waltham, MA, USA). Post-acquisition analysis was assessed using FlowJo software.

### Immunocytochemical staining of cultured cells

Cultured cells were mounted in slides and fixed with 4% PFA for 20 min at 4 °C and then were washed three times with 10 mM PBS (pH 7.4). Cells were permeabilized using 0.3% Triton X-100 for 20 min. Non-specific binding was blocked by incubating fixed cells with 5% BSA in PBS for 1 h at room temperature. Corresponding primary antibodies were diluted in BSA 1% and incubated overnight at room temperature. After washing, cells were incubated with secondary antibodies in BSA 1% for 1 h at room temperature. DAPI was used for nuclei staining.

### Intravenous injection of bone marrow mast cells into ALS mice

Bone marrow-derived mast cells (BMMC) cultures were obtained from SOD1^G93A^ symptomatic mice following the protocol previously described by Heig et al. 1994 [20]. Briefly, animals were euthanized by overdosing with ketamine/xylazine, and long bones were exposed. The bone marrow was flushed from the tibia and femur and collected in a 15 mL tube. After centrifugation for 10 min at 1000xg, pellet was resuspended in mast cell culture medium [RPMI 1640 medium/15% (vol/vol) FBS, sodium pyruvate (1 mM), β-mercaptoethanol (50 μM)] in presence of IL-3 (20 ng/mL) and SCF (20 ng/mL) freshly added every 48 h. The attached cells were discarded, non-adherent cells were resuspended and the culture medium was replaced every 48 h. After 2, 7, and 14 DIV, cells were collected and purity of BMMC culture was analyzed using flow cytometry and immunocytochemistry by staining with c-Kit antibody. Cell viability was assessed using the 0.4% trypan blue dye exclusion method before transplantation. Cells were stained with Cell Trace CFSE according to the manufacturer´s instructions (Thermo Fisher Scientific, #C34554) allowing the cell tracking after transplantation. Transplant cell concentration was adjusted to a final concentration of 5000 cells/μL (1 × 10^6^ cells/200 μL c-Kit+ BMMC) and were delivered intravenously by injection through the tail vein. Also, four Non-Tg mice were transplanted with c-Kit+ BMMC at a final concentration of 5000 cells/μL as control. The media-injected group received 200 μL of PBS, the same volume administered to the cell-transplanted mice. 48 h after injection, animals were euthanized by overdosing with ketamine/xylazine and spinal cords were processed for immunohistochemical analysis.

To determine whether masitinib could influence MCs trafficking and accumulation in the spinal cord, eight transgenic mice at 140 days old were randomly divided into the masitinib or vehicle-treated groups. Masitinib mesylate (AB1010) freshly prepared in drinking sterilized water was administrated daily at a dosage of 50 mg/kg/d using a curved stainless steel gavage needle with a 2-mm ball tip. Mice (*n* = 4 per group) were treated for 10 days. On day 8, MC precursors were intravenously injected as previously described. On day 10, Evans Blue (EB) extravasation protocol was performed. Briefly, animals were intracardially perfused with 4% PFA/1% EB under 90% ketamine/10% xylazine anesthesia and euthanized. Spinal cords were processed for immunohistochemistry analysis. Far-red fluorescence EB emission was detected by confocal microscopy.

### Analysis of microvasculature

To analyze vascular abnormalities in SOD1^G93A^ symptomatic mice, using a stereological approach, the number of string vessels and vessel sprouts stained with collagen type I antibody [55] were assessed as previously described [15, 22]. The counting was performed only in the area that surrounds motor neurons within a radius of 150 μm of the soma in the ventral horn of the lumbar spinal cord. The counting was carried out using maximum-intensity projection confocal microphotographs with a magnification of 63x. At least fifteen sections per spinal cord were analyzed (*n* = 4). Image J software was used for analysis. EB extravasation analysis was performed as previously described [23]. Animals were intracardially perfused with 4% PFA/1% EB under 90% ketamine/10% xylazine anesthesia. Then, the spinal cords were dissected, maintained overnight in 4% PFA, and cryopreserved in 30% sucrose (Sigma, St. Louis, MO, USA) at 4 °C. After 72 h, tissue was embedded in Tissue-Tek (Sakura), and 25 μm thick slices were sectioned using a Leica cryostat and collected on gelatin-coated slides. Then, far-red fluorescence EB emission was detected by confocal microscopy. EB analysis was performed either alone or after immunostaining with other markers. Quantitative analysis of perivascular EB extravasation was measured as the intensity in a 5 μm radius surrounding blood vessels using Image J software.

### Antibodies used

Primary antibodies: 1:250 mouse monoclonal anti-chymase (Abcam, #ab2377) 1:200 mouse monoclonal anti- tryptase (Abcam, #ab2378), 1:200 goat polyclonal anti-chymase (Abcam, #ab111239), 1:100 rat monoclonal anti-c-Kit (biotin) (Abcam, #ab25022), 1:300 rabbit polyclonal anti-collagen I (Abcam, #ab34710), 1:200 rabbit polyclonal anti- COX2 (Abcam, #ab15191), 1:200 mouse monoclonal anti-βIII-Tubulin (Millipore, #MAB1637), 1:100 rabbit polyclonal anti-SCF (Fisher Scientific, #PA520746), 1:400 mouse monoclonal anti-GFAP (Sigma, #G3893) and 1:100 rabbit polyclonal anti-CD45-PerCP (BioLegend, #103130). Secondary antibodies: 1:500 goat anti-rabbit- AlexaFluor488 or AlexaFluor546 (Thermo Fisher Scientific, #A11035 or #A11034), 1:500 goat anti-mouse-AlexaFluor488, AlexaFluor546 or AlexaFluor633 (Thermo Fisher Scientific, #A11029, #A11030, or #A21052), 1:500 donkey anti-goat- AlexaFluor488 (Thermo Fisher Scientific, #A11055) and 1:500 Streptavidin- AlexaFluor633 (Thermo Fisher Scientific, #S21375). NeuroTrace 530/615 red fluorescent Nissl stain (Thermo Fisher Scientific, #B34650) was also used for neuronal visualization.

### Fluorescence imaging

Fluorescence imaging was performed with a laser scanning Zeiss LSM 800 confocal microscope with either a 25 × (1.2 numerical aperture) objective or 63 × (1.3 numerical aperture) oil- immersion objective using Zeiss Zen Black software. Maximum intensity projections of optical sections were created with Zeiss Zen software.

### Bright-field imaging

DAB immunohistochemical imaging was acquired using a Zeiss LSM 800 microscope connected to an Evolution LC Color camera using 63 × (1.3 numerical aperture) oil- immersion objective using Zeiss Zen Black software.

### Statistical analysis

Quantitative data were expressed as mean ± s.e.m. For statistical analysis two-tailed Mann–Whitney test, Kruskal–Wallis followed by Dunn’s multiple-comparisons test, one-way ANOVA, or two-tailed unpaired t-test were used, with *p* < 0.05 considered significant. GraphPad Prism 7.03 software was used for statistical analysis.

## Results

### Mast cells accumulate nearby spinal motor neurons in ALS

**Fig. 1:**
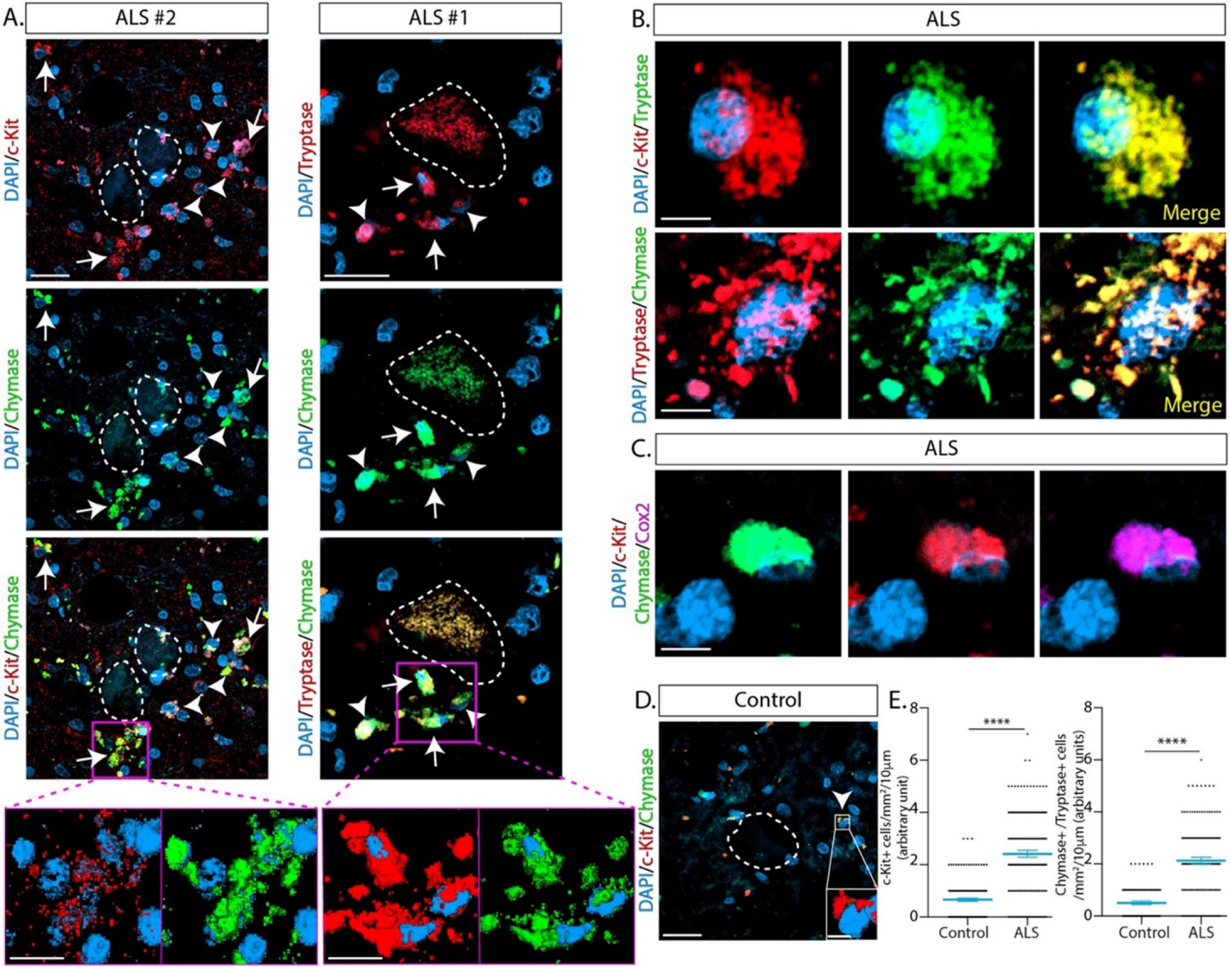
Mast cells accumulate in the surrounding of degenerating motor neurons in the spinal cord of ALS patients. **A** Representative immunohistochemical confocal images of mast cells infiltrating into the ventral horn of lumbar spinal cord of autopsy ALS subjects. Left panels show the expression of c-Kit+ (red) and chymase+ (green) mast cells displaying typical granular (white arrows) and non-granular (white arrowheads) morphology in the surroundings of degenerating motor neurons (white lines). The right panels show the co-expression of chymase (green) and tryptase (red) in mast cells. Magenta squares show high magnification 3D reconstructions of both, small non-granular and degranulating cells. **B** Representative high magnification confocal phenotyping of mast cells in the spinal cord of ALS subjects. Upper panels show a granular mast cell co-expressing c-Kit (red) and tryptase (green). Lower panels show a typical degranulating mast cell that co-express tryptase (red) and chymase (green). **C** Representative microphotograph of non-granular small mast cells with none or few granules that typically express c-Kit (red), chymase (green), and Cox-2 (magenta). **D** Representative confocal image of the immunohistochemical analysis performed in autopsy control donors, showing only a few small non-granular cells expressing c-Kit (white arrowhead) in the surroundings of motor neurons (white lines). The white square shows a high magnification 3D reconstruction of typical non-granular cells expressing c-Kit (red). **E** The graphs show the quantitative analysis of c-Kit+ (left) and tryptase+/chymase+ (right) cells in the area that surrounds motor neurons in the ventral horn of the lumbar spinal cord of ALS subjects compared to control donors. Quantitative data are expressed as mean ± s.e.m. Data were analyzed by a two-tailed Mann–Whitney test, with ***p < 0.0001. Scale bars: 20 µm and 10 µm in (**A**, **D**) and 5 µ in (**B**, **C**)

Previous studies on ALS have provided scarce information on MC phenotypes and precise localization in the postmortem patients’ spinal cords [19]. Thus, we examined five postmortem lumbar spinal cords from ALS subjects and four control donors by immunofluorescence confocal microscopy (Additional file 1: Table 1). In control donors, a scarce number of small cells expressing MC markers such as c-Kit, chymase, tryptase, or Cox-2 and bearing a granular cytoplasm, were identified in the grey matter surrounding motor neuron cell bodies (Fig. 1D and Additional file 1: Fig. 1). In comparison, all ALS specimens systematically displayed an increased density of cells bearing a MC phenotype (2.4 ± 1.6 vs. 0.6 ± 0.7 cells per analyzed field in ALS and controls, respectively), which preferentially accumulated in clusters nearby motor neuron somas (Fig. 1A and Additional file 1: Fig. 1). In ALS subjects, MCs displayed hypertrophy, granular morphology and frequent images of explosive degranulation, and strong staining with c-Kit, chymase, tryptase, and Cox-2 (Fig. 1B and Additional file 1: Fig. 1). In addition, a sub-set of c-Kit+ cells of small size and bearing few or no granules were also found in ALS specimens (Fig. 1D and Additional file 1: Fig. 1B). There was a fourfold increase in density of peri-neuronal c-Kit+ and chymase+/tryptase+ cells in the ALS spinal cord as compared with control donors (Fig. 1E and Additional file 1: Fig. 1).

### c-Kit+ mast cells accumulate in the degenerating spinal cord of ALS mice and rats

**Fig. 2:**
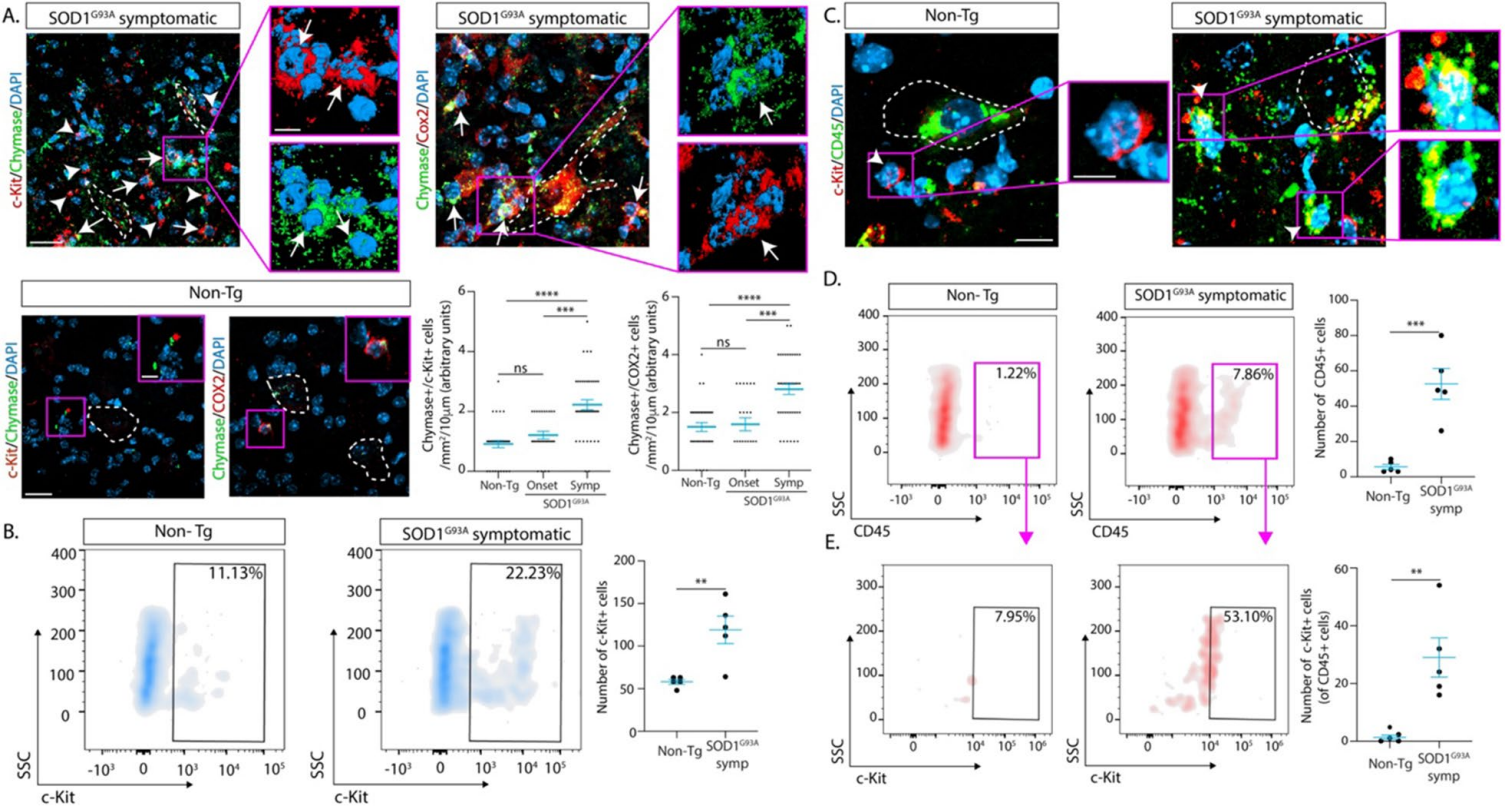
Mast cells accumulate in the surrounding of motor neurons in the spinal cord of SOD1^G93A^ mice. **A** Representative confocal immunohistochemical images showing the co-expression of c-Kit+/chymase+ (left upper panels), and chymase+/Cox-2+ (right upper panels) mast cells in the surroundings of degenerating motor neurons (white dotted lines) in the ventral horn of the symptomatic SOD1^G93A^ lumbar spinal cord. Mast cells display two phenotypes, typically bearing granular morphology with images of explosive degranulation (white arrows) and small rounded cells with few or no granules (white arrowheads). Magenta squares show high magnification 3D reconstructions of both, small non-granular cells, and mast cells showing an irregular shape corresponding to an explosive degranulating state (white arrows). Lower panels to the left show representative confocal images of mast cell markers staining in non-transgenic littermates, where only small cells with non-apparent granules or degranulation were observed (magenta squares). The graphs to the right show the quantitative analysis of chymase+/c-Kit+ and chymase+/Cox-2+ mast cells in the ventral horn of the lumbar spinal cord of SOD1^G93A^ symptomatic mice compared to Non-Tg littermates. **B** Flow cytometry analysis of the c-Kit+ cell populations of the spinal cord of Non-Tg and SOD1^G93A^ symptomatic mice (150d). The images to the left show the representative density plots of c-Kit expression. The graph to the right shows the quantitative analysis between conditions. Note that a statistically significant increase of c-kit expression is observed in symptomatic ALS mice when compared with controls. **C** Representative confocal images analyzing cells that co-express c-Kit (red) and CD45 (green) in the surroundings of motor neurons (white dotted lines) in both Non-Tg (left panel) and symptomatic SOD1^G93A^ littermates (right panel). The magenta squares show a high magnification analysis of c-Kit+/CD45+ cells in both conditions. Note the presence of few granules in small cells in the symptomatic condition, while significantly fewer smaller cells with no granules are observed in Non-Tg mice. **D** Flow cytometry analysis of c-Kit+/CD45+ cell population in the spinal cord of Non-Tg and symptomatic SOD1^G93A^ mice. Upper panels show representative density plots of CD45 expression, where a significant increase is observed in the ALS condition. The lower panels show representative density plots of c-Kit expression within the CD45 population previously analyzed. Graphs to the right show the quantitative analysis for CD45 and CD45/c-Kit respectively. Quantitative data are expressed as mean ± s.e.m. Data were analyzed by Kruskal–Wallis followed by Dunn´s multiple comparison test (**A**) and two-tailed unpaired t-test (**B**, **D**, **E**), with **p < 0,01, ***p < 0.001 and ****p < 0.0001 considered significant. *n* = 4 animals/condition. Scale bars: 20 μm and 5 μm in (**A**) in low and high magnification respectively, 10 μm and 5 μm in (**C**), in low and high magnification respectively

Next, we examined MC accumulation in the spinal cord of ALS murine models as this is still unknown. As shown in Fig. 2 and Additional file 1: Fig. 2 and 3, cells expressing c-Kit, chymase, and/or Cox-2 accumulated nearby degenerating motor neurons in symptomatic SOD1^G93A^ mouse and rat lumbar spinal cords, as compared with significantly fewer cells observed in SOD1^G93A^ mice and rats at the onset of the disease as well as in Non-Tg littermates (Fig. 2 and Additional file 1: Fig. 2 and 3). The predominant MC phenotype in the symptomatic SOD1^G93A^ mouse spinal cord corresponded to small cells bearing few granules and scarce images of explosive degranulation. Metachromasia following staining with toluidine blue that typically identifies MCs in epithelial or connective tissues [41] was not observed in MCs from the murine or human spinal cord (Additional file 1: Fig. 4). Quantitative analysis showed that the number of c-Kit+/chymase+ and c-Kit+/Cox-2+ MCs outnumbered by 2- and fourfold, respectively, the number of MCs in non-transgenic animals (Fig. 2A and Additional file 1: Fig. 2). To quantitatively analyze the c-Kit+ MC population, we performed flow cytometry analysis of the spinal cord. As shown in Fig. 2B, there was a twofold increase in the number of c-Kit+ cells in the symptomatic spinal cord of SOD1^G93A^ mice relative to Non-Tg littermates.

Next, we performed flow cytometry analysis to further characterize the population of c-Kit+ cells expressing CD45 from the spinal cord of symptomatic SOD1^G93A^ mice. CD45 is a well-recognized surface receptor marker of hematopoietic cells[51] and tumoral MCs [45]. As shown in Fig. 2C, c-Kit+ cells co-expressing CD45 and bearing MC morphology were abundant near motor neurons in ALS mice. The quantitative analysis of CD45+ and CD45+/c-Kit+ populations in the spinal cord by flow cytometry showed that hematopoietic-derived cells were significantly increased by sixfold in the symptomatic SOD1^G93A^ spinal cord relative to Non-Tg, further suggesting the accumulation of blood-born c-Kit+ MC putative precursors into the degenerating spinal cord of ALS mice.

### Ex vivo generation of mast cells from the spinal cord of SOD1^G93A^ rats

**Fig. 3:**
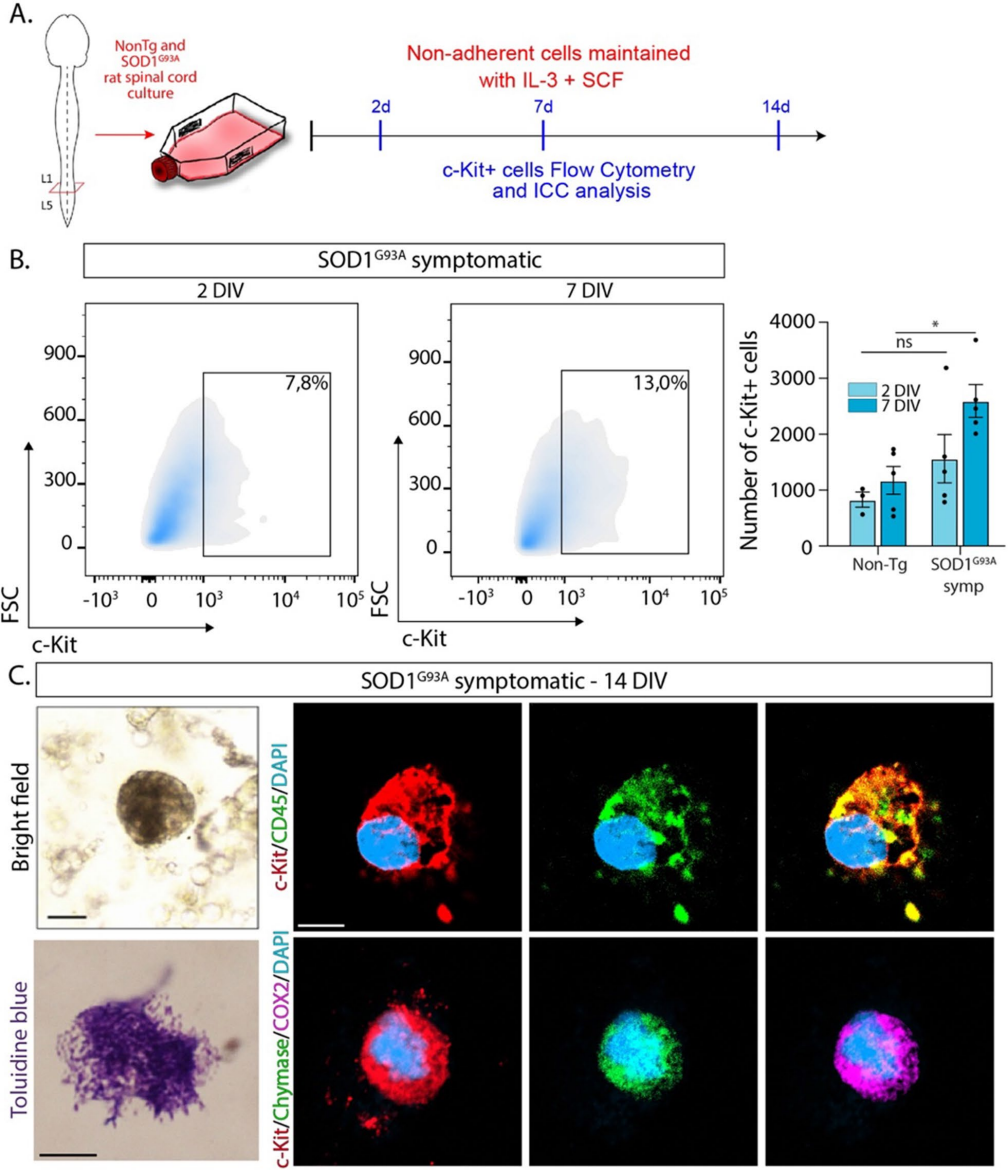
Ex vivo generation of c-Kit+ mast cells from the spinal cord of ALS rats. **A** The scheme shows the procedure followed to obtain primary cultures of mast cells from the adult spinal cord of symptomatic SOD1^G93A^ rats and Non-Tg littermates. Spinal cords were maintained in the presence of IL-3 (20 ng/mL) and SCF (20 ng/mL) for 2, 7, and 14 days in vitro (DIV), and then mast cell markers, c-Kit, chymase, Cox-2, and CD45 were analyzed by flow cytometry and immunocytochemistry. **B** Flow cytometry analysis of c-Kit+ cultured mast cells after 2 and 7 DIV. Representative density plots show the expression of c-Kit at 2 and 7 days. The graph to the right shows the quantitative analysis of c-Kit expression in cells cultured from Non-Tg and SOD1^G93A^ rats. **C** Representative cytological and confocal immunohistochemical images of mast cells isolated from symptomatic SOD1^G93A^ spinal cord. Left panels show representative bright-field images of non-adherent cells with showing typical granular mast cells (upper panel) and metachromatic granular mast cells stained with toluidine blue (lower panel). Panels to the right show immunohistochemical phenotyping by confocal microscopy of cultured mast cells expressing typical markers such as c-Kit, CD45, chymase, and Cox-2 after 14 days in culture. Quantitative data are expressed as mean ± s.e.m. Data were analyzed by the Mann–Whitney test with *p < 0,05 considered significant. *n* = 4 animals/condition. Scale bars: 5 μm

In peripheral tissues, MCs are originated from circulating blood MC-committed c-Kit+ progenitors in a process regulated by local factors that regulate trafficking, proliferation, and differentiation of MCs [7, 36]. We hypothesized that in ALS, similar c-Kit+ MC precursors infiltrate and accumulate into the spinal cord, preserving the ability to differentiate into mature MCs. To test this mechanism, we have used the already validated primary cell cultures from the spinal cord of symptomatic SOD1^G93A^ rats and non-transgenic littermates, in which a large number of precursor cells remain in a non-adherent phase [28]. Such non-adherent spinal cord cells were maintained for 7 days in the presence of SCF and interleukin-3 (IL-3) (Fig. 3A), cytokines that promote MC differentiation from bone marrow progenitors [20] as shown in Additional file 1: Fig. 7 in SOD1^G93A^ rats. During the SCF/IL3 differentiation protocol, the number of c-Kit+ cells in the spinal cord cultures increased from 8% after 2 days in culture to 13% at day 7 (Fig. 3B). Cytological analysis showed numerous images of fully differentiated, granular, metachromatic MCs expressing c-Kit, CD45, chymase, and Cox-2 (Fig. 3C), further indicating the accumulation of MC precursors in the ALS spinal cord. In comparison, spinal cord cultures from non-transgenic littermate rats maintained with SCF/IL-3 yielded a low number of c-Kit+ cells (Fig. 3 and Additional file 1: Fig. 8).

### Circulating c-Kit+ mast cell precursors engraft into the motor neuron-vascular niche

**Fig. 4:**
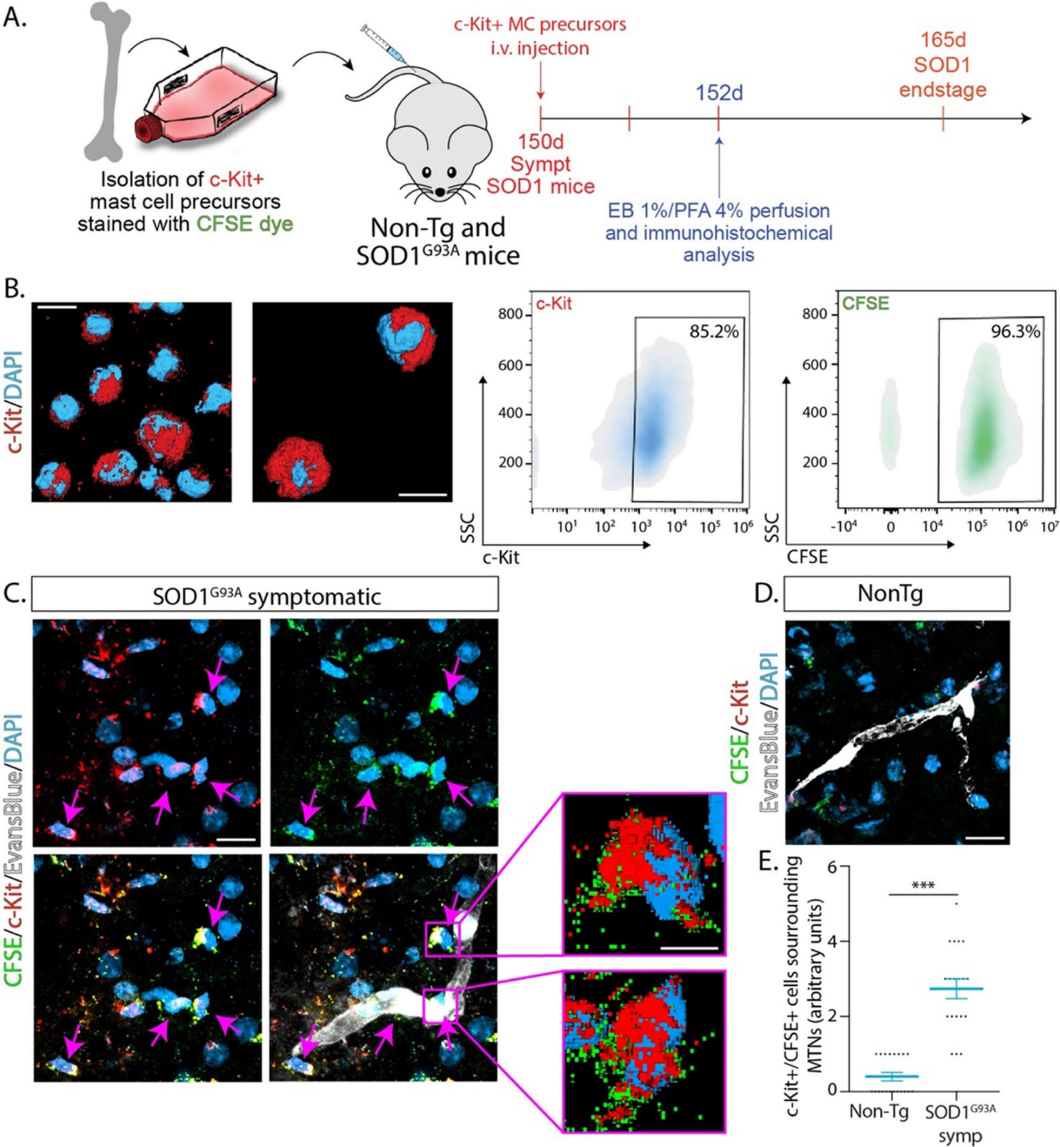
c-Kit+ mast cell precursors engraft into the degenerating spinal cord of SOD1^G93A^ mice following i.v. administration. **A** The scheme shows the isolation and culture of mast cells precursors from the bone marrow of Non-Tg mice and the protocol followed for the i.v. injection c-Kit+ mast cell precursors stained with the CFSE cell tracer in Non-Tg and symptomatic SOD1^G93A^ littermates at 150 days. **B** Left panels show representative 3D confocal images of c-Kit immunostaining of mast cells precursors. Right panels show the flow cytometry density plot analysis c-Kit+ (red—left panel) precursors stained with CFSE+ (green—right panel). **C** Representative confocal images showing infiltration of c-Kit+/CFSE+ mast cells precursors (magenta arrows) into the symptomatic SOD1^G93A^ spinal cord parenchyma following 48 h after i.v. administration. c-Kit+/CFSE+ cells engrafted the parenchyma in the surroundings of blood vessels assessed by EB systemically perfused after euthanasia (white). Magenta squares show high magnifications 3D reconstructions of typical c-Kit+/CFSE+ precursor cells. **D** Representative confocal image of the co-expression of c-Kit and CFSE in the spinal cord of i.v. injected Non-Tg mice. None of few cells were observed in the spinal cord parenchyma of controls. **E** The graph shows the quantitative analysis of c-Kit+/CFSE+cells in Non-Tg and SOD1^G93A^ mice. Quantitative data are expressed as mean ± s.e.m. Data were analyzed by two-tailed Mann–Whitney test, with***p < 0.001 considered significant. Scale bars: 10 μm (**A**and **B**) and 5 μm in (**A**)

Next, we explored whether peripheral c-Kit+ MC precursors isolated from the bone marrow of non-transgenic mouse donors and labeled with the cell tracer CFSE could infiltrate and engraft the spinal cord parenchyma following i.v. injection. Before transplantation, over 85% of MC precursors were c-Kit+ and CFSE+ (Fig. 4B). By 48 h following i.v. injection of 1 × 10^6^ million MC precursors into symptomatic SOD1^G93A^ recipient mice (150 days old), numerous CFSE+/c-Kit+ MC precursors were identified in the ventral horn in close association with microvascular elements (Fig. 4C). In comparison, no apparent or few CFSE+ cells were observed in the spinal cord of recipient non-transgenic mice, suggesting active trafficking of c-Kit+ MC precursors through the disrupted microvasculature in ALS mice.

### Mast cells associate with abnormal perineuronal microvascular elements

**Fig. 5:**
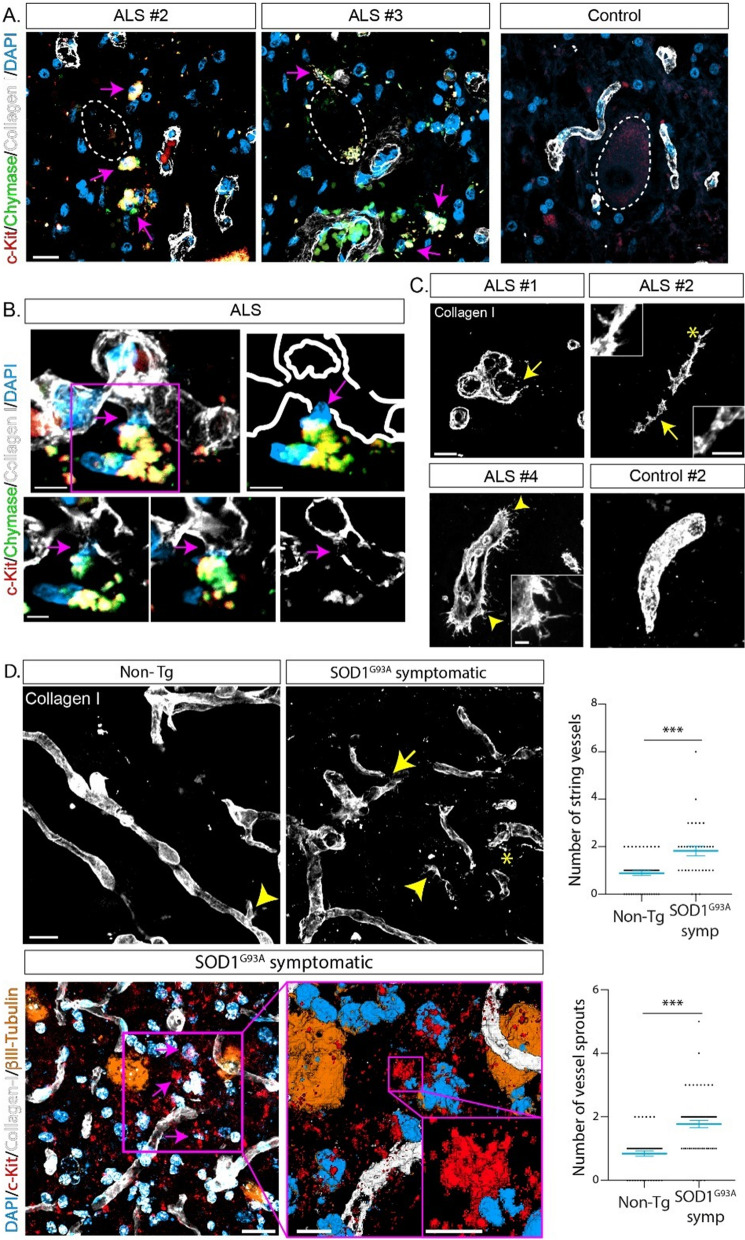
Mast cells are associated with altered microvascular elements in ALS patients and mice. **A** Representative confocal microphotographs of c-Kit+ (red)/chymase+ (green) mast cells (magenta arrows) associated with microvascular elements stained with Collagen-I (white) in the surroundings of degenerating motor neurons (white dotted lines) in the lumbar spinal cord of autopsy ALS subjects and control donors. Both small non-granular and degranulating mast cells are observed in the ALS spinal cords (left and middle panel), as compared with few small rounded cells present in the spinal cord of control donors (right panel). **B** High magnification confocal analysis of c-Kit+/chymase+ mast cells associated with pathological blood vessels. Note that granular mast cells seem to emerge from a blood vessel stained with collagen-I into the parenchyma of the spinal cord. High magnification analysis (magenta square) is shown in the right upper panel where the boundaries of the blood vessel were depicted only as a white solid line. The close contact between damaged blood vessels and mast cells is indicated by the magenta arrow. The three panels below show orthogonal views of the z stack, to illustrate the damage observed in the blood vessels in contact with mast cells (magenta arrows). **C** Representative confocal analysis of pathological features of microvasculature in the ALS lumbar spinal cord. Frequent morphological abnormalities as observed by collagen-I interruptions (yellow arrow ALS #1), strings (yellow asterisks ALS #2), and sprouts (yellow arrowheads ALS #4). Alterations in the microvasculature are not observed in control donors (control #2). **D** Representative confocal characterization of abnormal microvascular elements stained with collagen-I (white) in the spinal cord of symptomatic SOD1^G93A^ mice but not in Non-Tg littermates (upper panels). Lower panels show the association of c-Kit+ mast cells with the motor neuron-vascular niche. βIII-Tubulin antibody was used to stain motor neurons (orange) and collagen-I to stain blood vessels (white). The magenta squares show a 3D high magnification analysis of the niche and c-Kit+ mast cells (red—magenta arrows) respectively. The graph to the right shows the quantitative analysis of the number of string vessels and vessel sprouts in SOD1^G93A^ symptomatic compared to Non-Tg littermates. Data are expressed as mean ± s.e.m. For statistical analysis two-tailed Mann–Whitney test was used, with ***p < 0.0001 considered significant. *n* = 4 animals/condition. Scale bars: 20 μm in (**A**and **C**), 10 μm in (**D**), and 5 μm in (**B**)

To test whether increased vascular permeability to c-Kit+ MC precursors was due to disruption of microvascular elements previously described in ALS [16, 17, 23], we analyzed microvascular pathology in the discreet compartment surrounding spinal motor neuron cell bodies. Figure 5A shows that in the spinal cord of ALS patients, tryptase+/chymase+ and c-Kit+ MCs were localized close to microvascular elements stained with type-I collagen (Fig. 5A and Additional file 1: Fig. 9). Moreover, microvasculature associated with MCs showed frequent morphological abnormalities as observed by collagen-I interruptions, strings, and sprouts (Fig. 5C and Additional file 1: Fig. 9), which resemble abnormal microvasculature in other neurodegenerative conditions such as Alzheimer disease [15, 22]. In comparison, the motor neuron-vascular niche in control donor sections displayed a closer contact between the neuronal cell bodies and capillaries, absence of vascular abnormalities, and none or a scarce number of associated MCs (Fig. 5A, C, Additional file 1: Fig. 9). In addition, peri-neuronal vascular elements in ALS mice displayed morphological abnormalities like those described in ALS subjects (Fig. 5D).

### c-Kit+ mast cells interact with astrocytes and motor neurons expressing stem cell factor

**Fig. 6:**
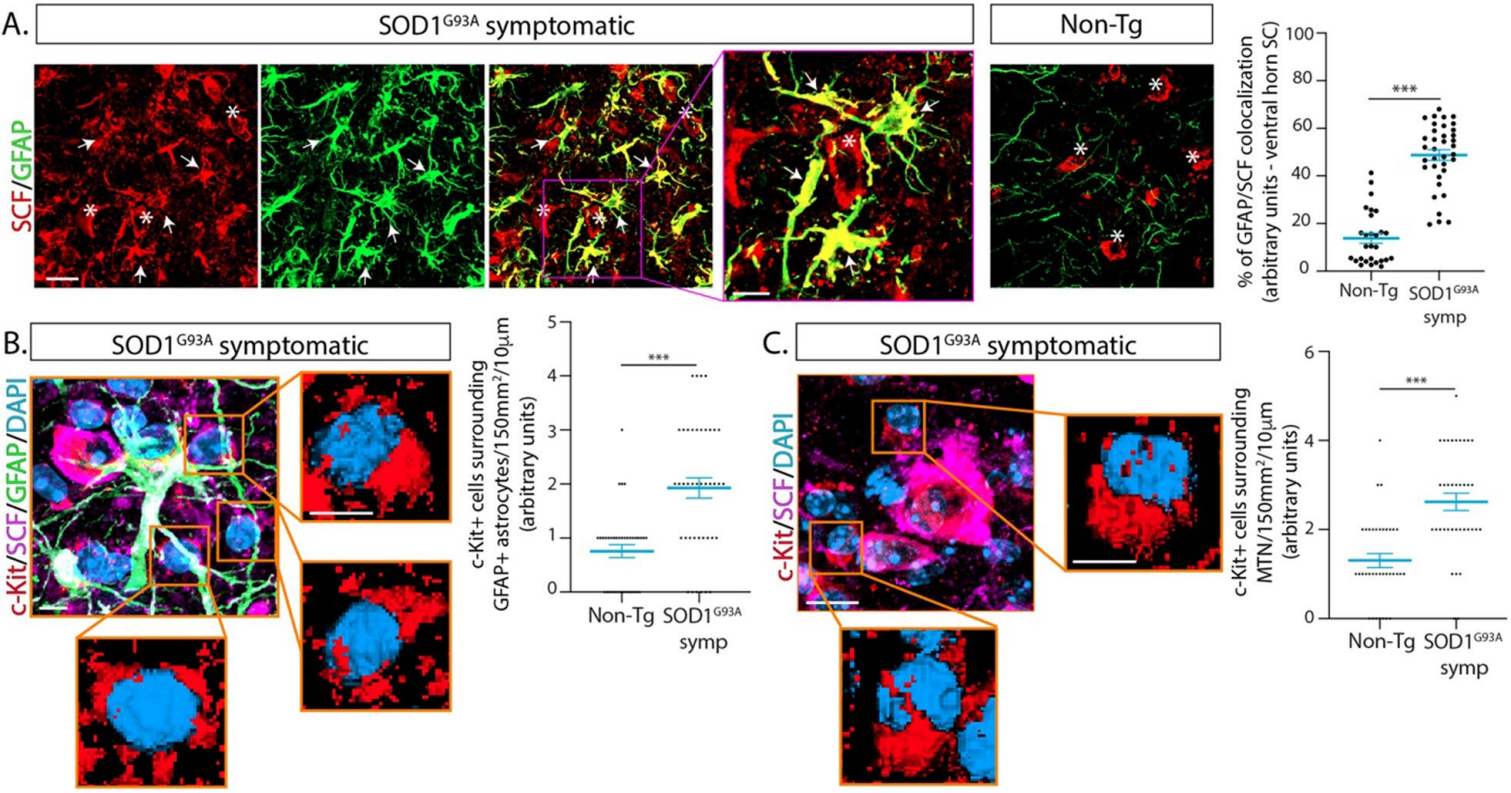
Mast cells interact with astrocytes and motor neurons expressing Stem Cell Factor in ALS mice. **A** Representative immunohistochemical confocal images showing the characterization of the c-Kit ligand expression, SCF, in the lumbar spinal cord of Non-Tg and symptomatic SOD1^G93A^ mice. Note the significant upregulation of SCF in reactive GFAP+ astrocytes (white arrows) in symptomatic ALS mice as compared with Non-Tg littermates. Motor neurons (white asterisks) express similar levels of SCF in both Non-Tg and SOD1^G93A^ mice. The magenta square shows a high magnification analysis of a SCF+ motor neuron surrounded by reactive astrocytes co-expressing high levels of GFAP and SCF (colocalization in yellow). The graph to the right shows the quantitative analysis of the co-localization of SCF and GFAP in spinal cord sections of SOD1^G93A^ as compared with Non-Tg mice. **B** High magnification confocal analysis of c-Kit+cells lacking granules that spatially associate with SFC + /GFAP+ reactive astrocytes. Squares show higher magnification 3D images of c-Kit+ cells (red) that surround reactive astrocytes. The graph shows the quantitative analysis of c-Kit+ cells surrounding SCF+ astrocytes. **C** High magnification confocal analysis of c-Kit + cells that closely interact with SFC+ motor neurons. Squares show higher magnification 3D images of c-Kit+ cells (red). The graph shows the quantitative analysis of c-Kit+ cells surrounding SCF+ motor neurons. Data are expressed as mean ± s.e.m. For statistical analysis two-tailed Mann–Whitney test was used, with ***p < 0.0001 considered significant. *n* = 4 animals/condition. Scale bars: 20 μm (A) and 10 μm and 5 μm (**B**, **C**)

Because c-Kit receptor is activated by the cytokine SCF which mediates MC chemoattraction and differentiation [36], we explored whether c-Kit+ MCs were associated with increased expression of SCF in the ALS motor neuron-vascular niche. As shown in Fig. 6A, SCF was strongly upregulated in reactive astrocytes in the ventral horn of symptomatic SOD1^G93A^ mice but not in controls. The number of reactive astrocytes co-expressing GFAP and SCF showed a 5 to sixfold increase relative to non-transgenic mice (Fig. 6A). SCF+ astrocytes spatially interacted with c-Kit+ MCs, suggesting astrocytic SCF may facilitate MC precursors infiltration and differentiation (Fig. 6B). In addition, spinal motor neurons from both non-transgenic and SOD1^G93A^ mice appeared to express intense staining for SCF (Fig. 6C, Additional file 1: Fig. 10), indicating an alternative cellular source of the cytokine.

### Post-paralysis c-Kit inhibition with masitinib prevents MCs trafficking and accumulation in the motor neuron-vascular niche

**Fig. 7:**
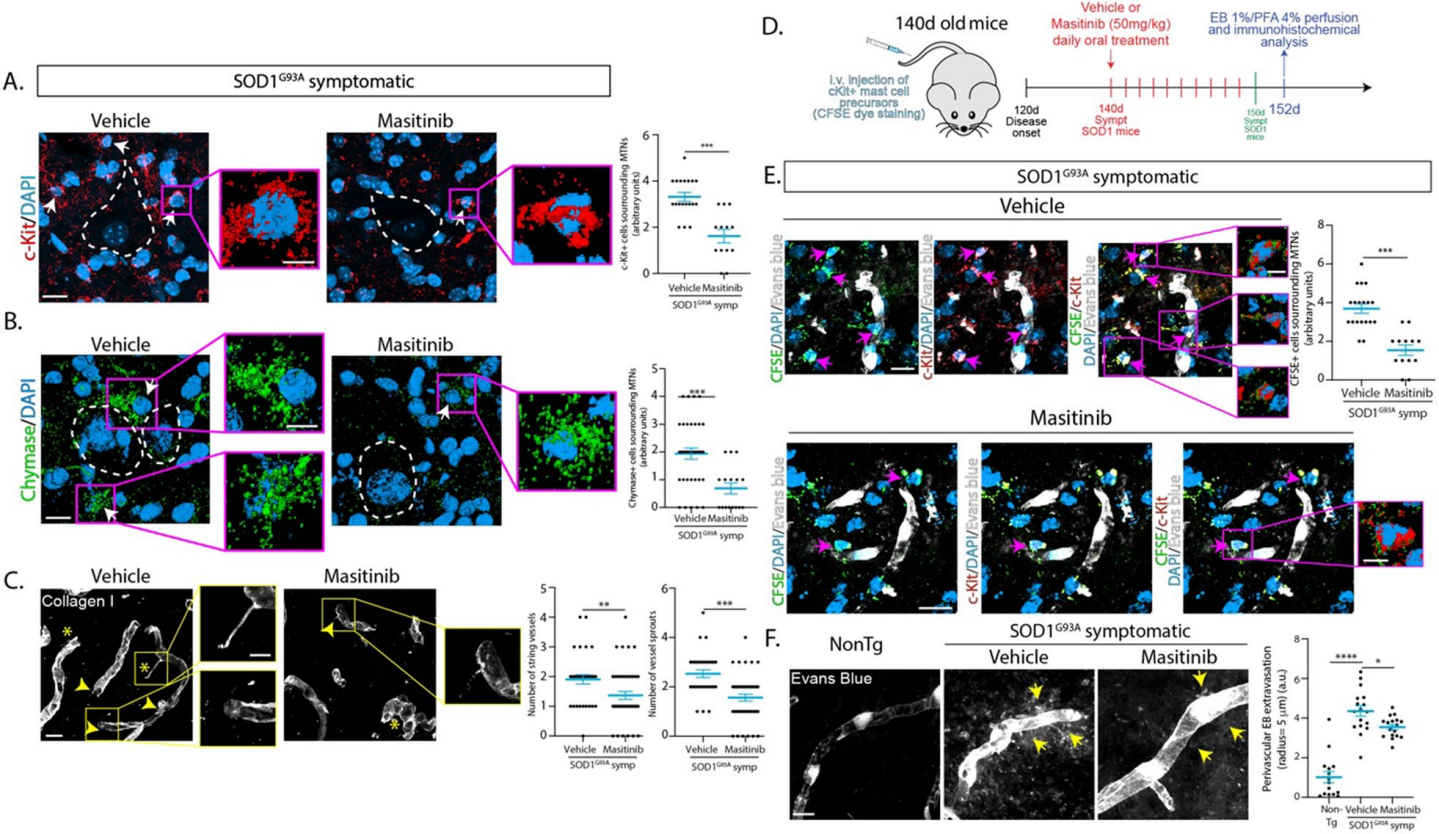
Post-paralysis masitinib treatment prevents mast cell accumulation and trafficking into the spinal cord of ALS mice. SOD1^G93A^ 140d old symptomatic mice were orally (gavage) daily treated with 50 mg/Kg of masitinib (or vehicle) for 10 days and immunohistochemical analysis was performed to characterize the presence of mast cells in the lumbar spinal cord. **A** Representative confocal images show the analysis of c-Kit+ cells (red – white arrows) accumulated in the surroundings of motor neurons (white dotted lines) in the lumbar spinal cord of SOD1^G93A^ mice after vehicle or masitinib treatment. Magenta squares show high magnification 3D images of typical c-Kit+ mast cells in both conditions. The graph shows that masitinib treatment significantly prevents the accumulation of c-Kit+ cells in the parenchyma of the spinal cord when compared with vehicle-treated mice. **B** Representative confocal images that show the analysis of chymase+ mast cells (green—white arrows) associated with motor neurons (white dotted lines) in the spinal cord of vehicle- and masitinib-treated SOD1^G93A^ mice. Magenta squares show high magnification 3D images of typically degranulating mast cells in proximity to motor neurons. Quantitative analysis in the graph shows that masitinib treatment significantly prevents the accumulation of degranulating chymase+ cells in the spinal cord. **C** Confocal microscopy analysis of the microvasculature pathology in SOD1^G93A^ treated with vehicle or masitinib. The number of vessels strings and vessels sprouts were quantified between groups in Collagen-I stained spinal cords. The graph shows that after 10 days of treatment with masitinib, there is a significant reduction of microvasculature alterations. **D** The scheme shows the experimental protocol for the i.v. delivery of c-Kit+/CFSE+ cells, after 10 days of the vehicle or masitinib treatment. **E** After 10 days of vehicle (upper panels) or masitinib (lower panels) treatment, c-Kit+ mast cells precursors (red) stained with CFSE dye (green) were i.v. injected in symptomatic 150d old SOD1^G93A^ mice. c-Kit+/CFSE+ precursors (magenta arrows) were analyzed in the periphery of blood vessels stained with EB dye systemically perfused after euthanasia (white). Magenta squares show high magnification 3D images of typical c-Kit+/CFSE+ cells infiltrating the spinal cord. Masitinib treatment significantly reduced the number of c-Kit+ cells infiltrating the spinal cord parenchyma of SOD1^G93A^ mice. The graph shows the quantitative analysis of c-Kit+ /CFSE+ cells in the surroundings of motor neurons. **F** Non-Tg, and vehicle- and masitinib-treated SOD1^G93A^ mice were systemically perfused after euthanasia with EB to measure the perivascular dye extravasation in the parenchyma of the spinal cord. Note the significant perivascular EB extravasation in SOD1^G93A^ vehicle-treated mice when compared to Non-Tg littermates. Masitinib significantly reduced perivascular extravasation after 10 days of treatment. The graph shows the quantitative analysis of perivascular EB extravasation among groups. Quantitative data are expressed as mean ± s.e.m.; data were analyzed by a two-tailed Mann–Whitney test (**A**–**C**, **E**) and one-way ANOVA (**F**) with *p < 0,05, ***p < 0.001 and ****p < 0.0001 considered significant. *n* = 4 animals/condition. Scale bars: 10 μm (low magnification panels) and 5 μm (insets)

Because masitinib potently inhibits the SCF/c-Kit pathway in MCs [11], we hypothesized that systemic administration of masitinib (50 mg/kg/d) in SOD1^G93A^ mice could prevent the trafficking and accumulation of MCs in the spinal motor neuron-vascular following i.v. injection. As shown in Fig. 7A, treatment with masitinib for 10 days significantly reduced by twofold the number of c-Kit+ and chymase+ MCs (Fig. 7B) in the lumbar motor neuron-vascular niche, respect to the vehicle-treated mice. Also, masitinib-treated animals showed a 30–40% reduction in microvascular abnormalities relative to vehicle-treated mice (Fig. 7C) and a 50% reduction in the number of CFSE-labeled c-Kit+ MC precursors infiltrating the spinal cord parenchyma (Fig. 7D). These results are consistent with a masitinib protective effect via c-Kit inhibition, preventing the trafficking of MC precursors and MC local differentiation in the motor neuron-vascular niche.

## Discussion

Here we report that the cellular niche comprised between motor neuron somas and nearby microvascular elements in the spinal cord of ALS subjects and murine models is the recipient of yet unrecognized clusters of MCs that are not observed in controls. Within this niche, we identified an array of c-Kit+ MC phenotypes, including typical degranulating MCs bearing specific markers such as chymase, tryptase, and Cox-2, but distinctively lacking the toluidine blue metachromasia characteristic of MCs in the periphery. MC degranulation denotes active secretion of inflammatory and vasoactive compounds with the potential to locally affect the neurovascular crosstalk [44]. In accordance, microvascular elements associated with MCs were severely altered in ALS subjects and murine models as compared with controls, suggesting a bidirectional causality underlying MC infiltration and defective BSCB permeability. Furthermore, we provide evidence that overexpression of the c-Kit ligand SCF in reactive astrocytes may drive the local chemoattraction and subsequent differentiation of MCs. In accordance, treatment of ALS mice with the c-Kit inhibitor masitinib, which has recognized anti-inflammatory and neuroprotective effects in ALS, significantly reduced MC number and trafficking as well as the microvascular pathology in ALS mice. Together, these findings provide the first description of disease-associated MC phenotypes in the ALS spinal cord and reveal novel potential interactions between MCs and the cellular components of the motor neuron-vascular niche.

It is well-established that MCs infiltrate the CNS-affected regions in neurodegenerative diseases, including ALS [19], contributing to neuroinflammation [43]. However, the interplay between MCs, motor neurons, and microvascular elements has remained unknown. In the periphery, MCs accumulate in increased number and MC degranulation correlates with paralysis progression in the ALS peripheral motor nerves and denervated NMJs [48, 49]. Similarly, here we found that MCs spatially interact with ALS-affected spinal cord motor neurons. All patients analyzed displayed remarkable differences with respect to the controls regarding the pattern of MC infiltrating the motor neuron-vascular niche, including (i) significantly increased MC density, (ii) frequent images of MC degranulation, and iii) increased number of perineuronal small non-granular c-Kit+ cells, which could correspond to MC precursors. The interaction between motor neurons and degranulating MCs anticipates complex and relevant functional crosstalk mediated by MCs secreted proteases, cytokines, trophic factors, and vasoactive mediators [34]. MCs also release nerve growth factor (NGF) species [29], potentially leading to pro-apoptotic signaling through 75-kD neurotrophin receptors (p75^NTR^) that are abnormally expressed in ALS-damaged motor neurons [37]. Thus, degranulating MCs in the subtle motor neuron-vascular compartment have the potential to trigger neuronal pathology and neuroinflammation, directly and indirectly.

The finding of MC clustering around defective peri-neuronal microvascular elements was associated with significant vascular abnormalities such as endothelial sprouts and string vessels, suggests a MC-mediated vascular pathology in ALS. Previous studies have shown impairment of microvascular endothelial cells and pericytes in the brain and spinal cord of rodent models of disease and ALS patients [16, 54], associated with compromised integrity of the blood–brain and blood-spinal cord barriers [17]. Similar microvascular aberrations have been described in Alzheimer’s disease [15], however, the mechanisms of microvascular pathology remain elusive. MCs play important roles in increasing microvascular permeability, in part through the release of chymase and tryptase that are known to degrade adhesion proteins complexes between endothelial cells [8] as well as the extracellular matrix proteins vitronectin, procollagen, type IV collagen [9, 30]. The release of histamine and Cox-2-synthesized prostaglandins by MCs may further increase microvascular increased permeability [1], facilitating the influx of peripheral inflammatory cells. In parallel, degranulating MCs may also promote angiogenesis via the secretion of several proteins including VEGF [35], a recognized neuroprotective trophic factor in ALS [2]. The absence of toluidine blue staining could indicate that mast cells have already degranulated, but does not necessarily mean they are unable to continue to secrete compounds that affect the disease. Thus, MC activation and degranulation in the motor neuron-vascular niche may have protective and regenerative effects in the early stages of ALS, then switching to deleterious pro-inflammatory and neurotoxic effects in more advanced stages of the disease.

The degenerating spinal cord in ALS murine models is characterized by an increased number of NG2 [25] and CD34 glial precursors [28], which contribute to extensive gliosis. However, the presence of MC precursors in the CNS affected regions in ALS remained unexplored. Here, we show evidence that fully differentiated MCs can be generated *ex-vivo* from the spinal cord of symptomatic ALS rats when non-adherent cells obtained in primary culture are maintained in the presence of IL-3 and SCF, conditions known to generate MCs from bone marrow [20]. We speculate MC precursors in the ALS spinal cord could correspond to the population of small non-granular c-Kit+ cells that are found in significant numbers in the motor neuron-vascular niche of both ALS subjects and murine models, suggesting that only a portion of c-Kit+ MC precursors trafficking from blood to the spinal cord readily differentiate into typical MCs containing granules. Moreover, we also show evidence that c-Kit+ MC precursors can traffic from the blood to the spinal cord following i.v. administration into symptomatic ALS mice. This trafficking was only observed in symptomatic ALS mice, indicating a prerequisite of permeable microvasculature. Thus, MCs precursors infiltration and subsequent differentiation into the spinal cord seem to be a complex process controlled by the integrity of BSCB and the overexpression of SCF in reactive astrocytes and neurons. In turn, MCs can also damage the BSCB suggesting a bidirectional pathogenic process that could be relevant for the integrity of the motor neuron-vascular niche.

We found that tyrosine kinase inhibition with masitinib downregulated the accumulation of MCs and trafficking of MC precursors into the motor neuron-vascular niche, which in turn resulted in a significant improvement of aberrant microvasculature. Because masitinib not only inhibits c-Kit but also kinases CSF-1R, PDGF-R, Lyn, and Fyn, the mechanism for this restorative effect could involve multiple pathways [11]. Neuroprotection by masitinib in ALS was originally linked to inhibition of tyrosine kinase receptor CSF-1R in microglia and aberrant glial cells that typically proliferate after paralysis onset [46, 47]. Subsequent studies have shown the drug also downregulates MCs, neutrophils, and macrophages in the ALS peripheral motor pathways [21, 48–50]. The present data show evidence that masitinib downregulates MCs infiltration and differentiation and prevents microvascular pathology by inhibiting SCF/c-Kit signaling, which could be fueled by the strong upregulation of SCF in reactive astrocytes in ALS mice [36].

This study has two main limitations. The first one is the small number of ALS subjects analyzed which prevents any correlation of mast cell infiltration with common genetic or ALS pathological features such as TDP-43 aggregates. The second limitation is the use of murine models based on overexpression of mutant human SOD1, which represent only a minor cause of familial ALS. However, the fact that mast cells and their precursors in the motor neuron-vascular niche are systemically found in all ALS subjects analyzed as well in the paralytic phase of animal models, suggest mast cells are an integral part of the neuroinflammatory response to motor neuron damage, as astrocytosis, microgliosis, and immune cell infiltration [6, 18, 33].

**Fig. 8:**
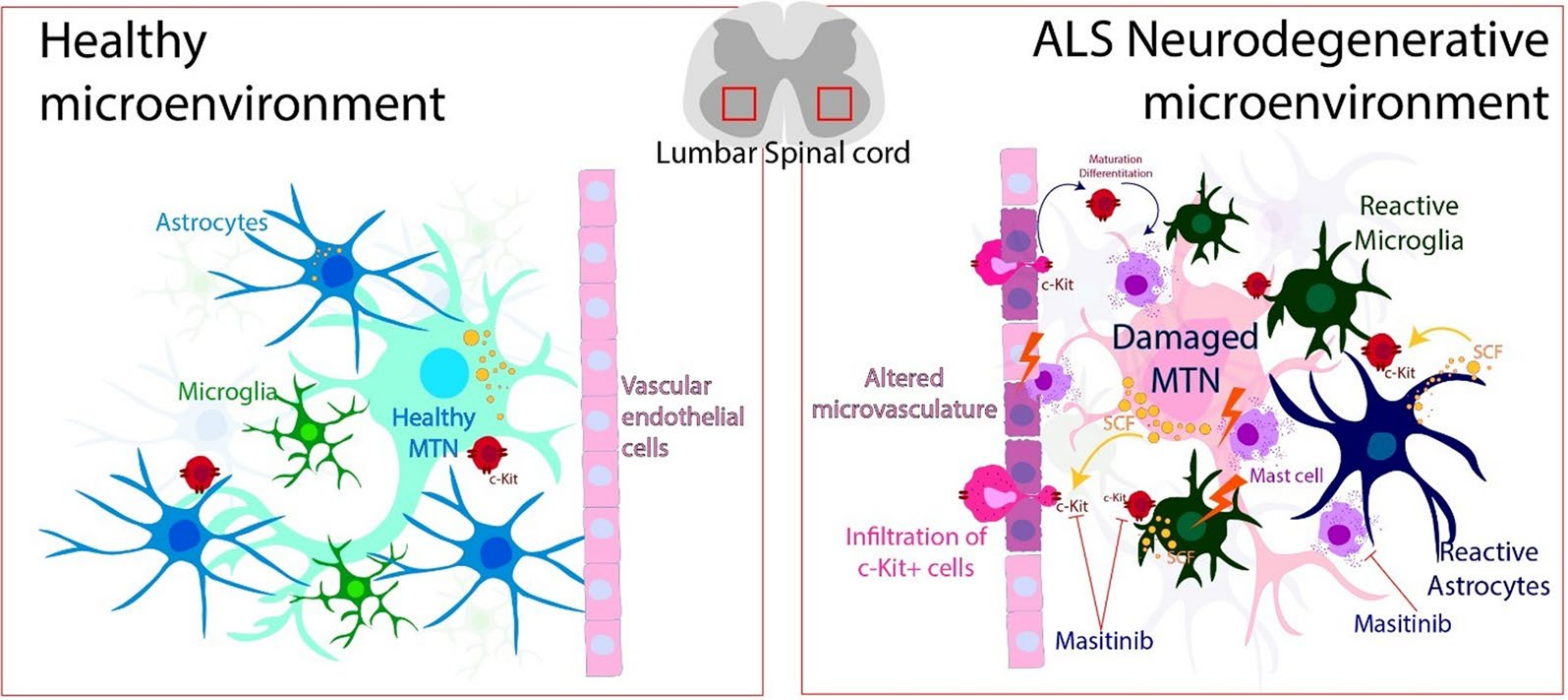
Schematic hypothesis about the pathogenic role of mast cells in ALS. c-Kit+ MC precursors infiltrate from the blood through altered microvascular elements that surround the degenerating/damaged motor neurons (MTN) and accumulate in the ALS spinal cord. The c-Kit ligand SCF expressed in reactive astrocytes and motor neurons promotes chemoattraction of c-Kit+ precursors that differentiate into MC bearing granular morphology and expressing typical MC markers exerting a potentially toxic effect on the microvasculature, glial cells, and motor neurons. Pharmacological inhibition of c-Kit reduces the MC number and the microvasculature pathology

Collectively, the present study shows for the first time that MCs and their c-Kit+ precursors are abundant in the motor neuron-vascular niche, representing a potential pathogenic cell type underlying ALS pathology. As summarized in Fig. 8, MCs appear to infiltrate the spinal cord through a mechanism wherein MC precursors traffic from the periphery across defective microvascular elements with subsequent local differentiation that is possibly driven by reactive astrocytes and motor neurons expressing high levels of the c-Kit ligand SCF. Thus, MCs appear to be relevant pro-inflammatory and microvascular effectors in ALS with the potential to be pharmacologically targeted by tyrosine kinase inhibitor drugs such as masitinib.

## Supplementary Information

**Additional file 1:** Supplementary Results with Figures 1–10 and Table 1.

## Data Availability

The authors confirm that the data supporting the findings of this study are available within the article and its Supplementary Material.

## Abbreviations

ALS: Amyotrophic lateral sclerosis
BBB: Blood brain barrier
BSCB: Blood spinal cord barrier
BMMC: Bone marrow mast cells
Cox-2: Cyclooxigenase 2
CSF-1R: Colony stimulating factor 1 receptor
EB: Evans Blue
IL-3: Interleukin-3
MCs: Mast cells
NMJ: Neuromuscular junction
PDGF-R: Platelet-derived growth factor receptor
SCF: Stem cell factor
