# Supplementary material for: The pathogenic role of c-Kit+ mast cells in the spinal motor neuron-vascular niche in ALS
Source: Acta Neuropathol Commun. 2021 Aug 13;9:136. doi: 10.1186/s40478-021-01241-3 (PMC8361844; doi:10.1186/s40478-021-01241-3)
Supplement: Supplementary file 1 — Additional file 1: Supplementary Results with Figures 1–10 and Table 1. [file 40478_2021_1241_MOESM1_ESM.pdf]

Additional File 1

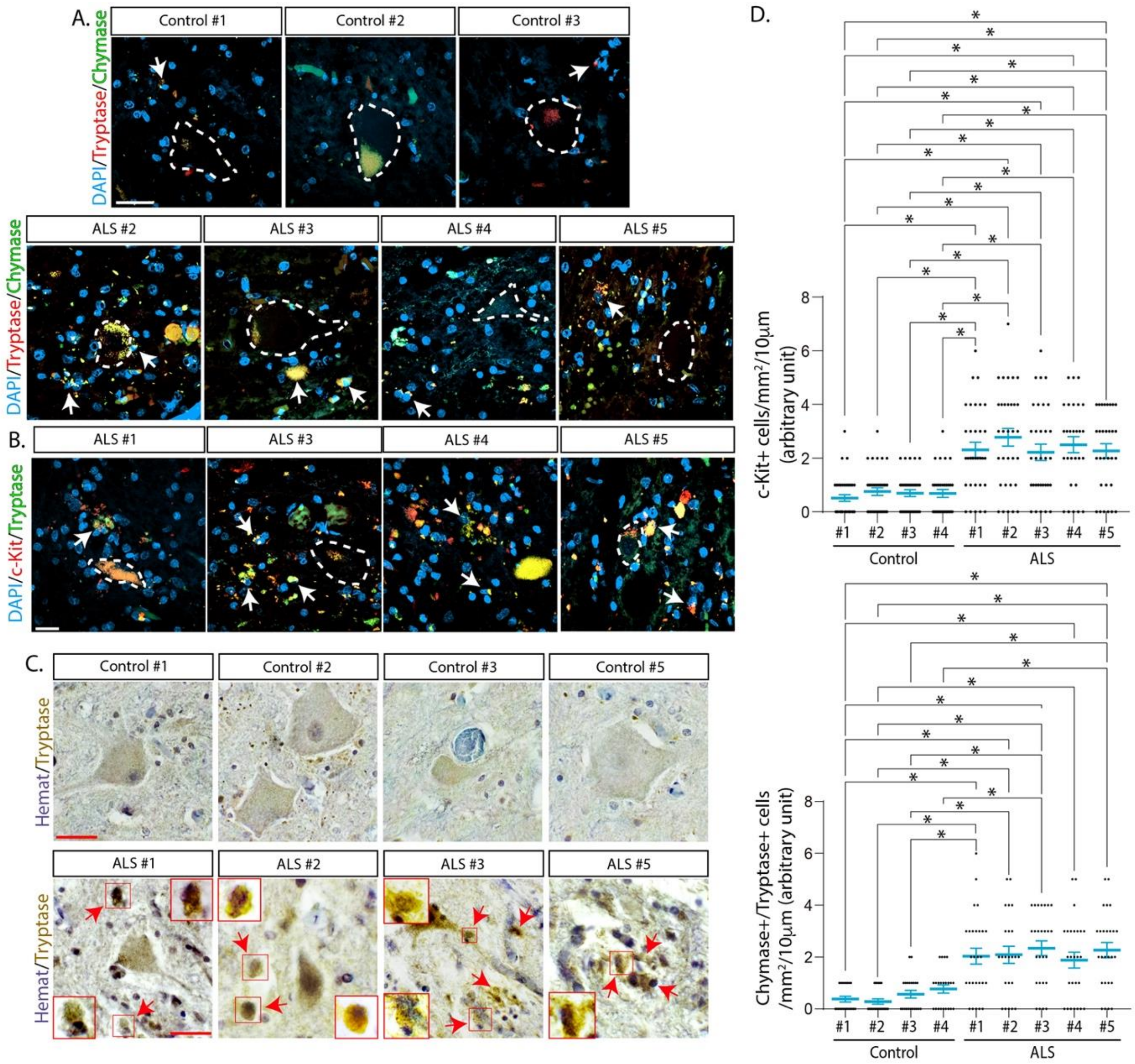

Figure 1. Mast cells accumulate in the surrounding of motor neurons in the spinal cord of ALS patients. **(A-B)** Representative confocal images of tryptase+/chymase+ **(A)** and c-Kit+/ tryptase+ **(B)** MCs infiltrating into the ventral horn of lumbar spinal cords of autopsy control and ALS patients. Arrows indicate cells stained with both markers (yellow). Motor neuron cell bodies are depicted in dotted lines. **(C)** Representative bright field microscopy images of tryptase (DAB)/Hematoxylin (Hemat) staining in autopsy ALS and control tissue. None or few tryptase+ mast cells were observed in control donors. Red arrows in lower panels show numerous MCs in the surroundings of motor neurons and microvasculature structures. Red squares show high magnification images of tryptase+mast cells. **(D)** Quantitative analysis of c-Kit+ (upper graph) and chymase+/tryptase+ (lower graph) cells in the area that surrounds motor neurons in the ventral horn of the lumbar spinal cord of ALS patients compared to control donors, individually. Only statistically significant comparisons are shown between control donors and ALS

subjects. Quantitative data are expressed as mean ± s.e.m. Data were analyzed by Kruskal-Wallis followed by Dunn's multiple-comparisons test. Scale bars: 20 μm.

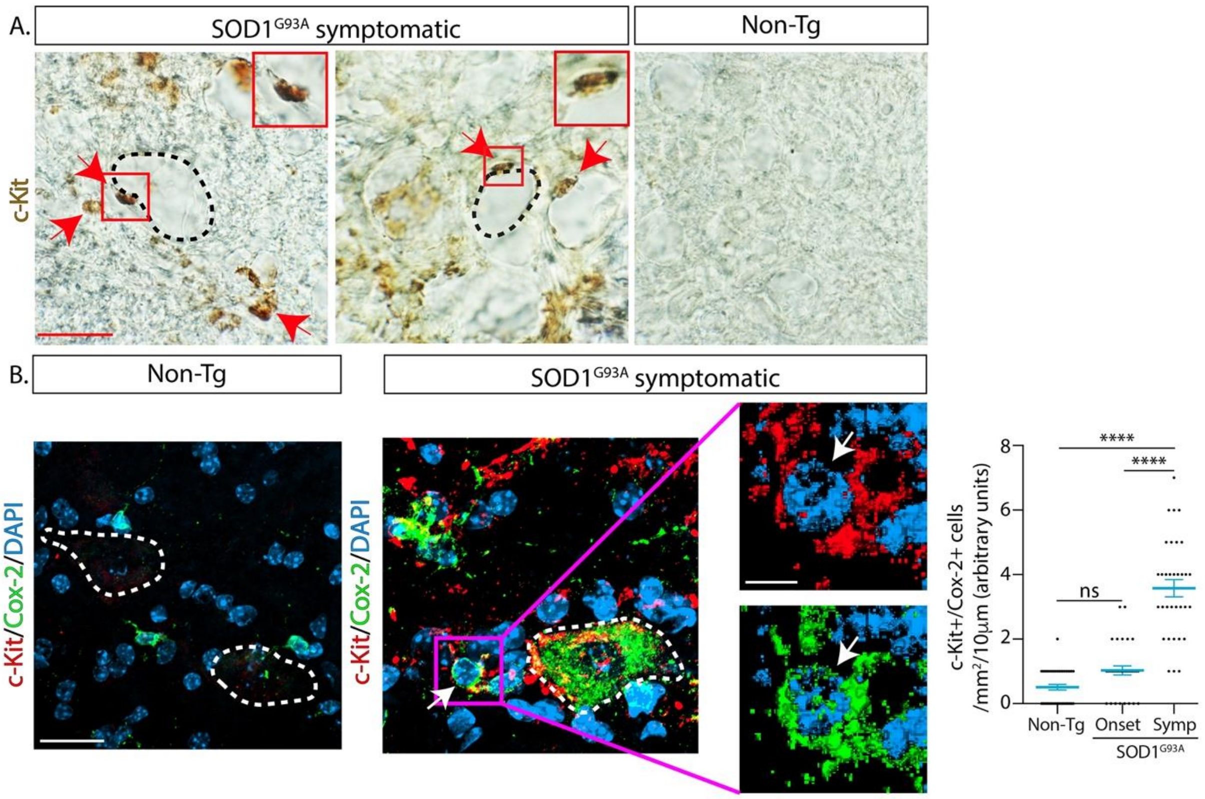

Fig. 2. c-Kit+/Cox-2+ mast cells in the spinal cord of ALS mice. **(A)** Representative bright-field microscopy images showing c-Kit (DAB) staining in the ventral horn of the spinal cord of symptomatic SOD1^{G93A} mice but not in Non-Tg mice. Red arrows show MCs in the surrounding of motor neurons (black dotted lines). Red squares show high magnification panels of c-Kit+ MCs. **(B)** Representative confocal images showing c-Kit+/Cox-2+ MCs in the surrounding of motor neurons (white dotted lines) in the ventral horn of the lumbar spinal cord of symptomatic SOD1^{G93A} mice and Non-Tg mice. Magenta square shows the area taken for 3D reconstructions of MCs. The graph to the right shows the quantitative analysis of c-Kit+/Cox-2+ MCs among conditions. Quantitative data are expressed as mean ± s.e.m. Data were analyzed by Kruskal-Wallis followed by Dunn´s multiple comparison test with ****p<0,0001 considered significant. n=4 animals/condition. Scale bars: 20 μm (low magnification panels) and 5 μm (insets).

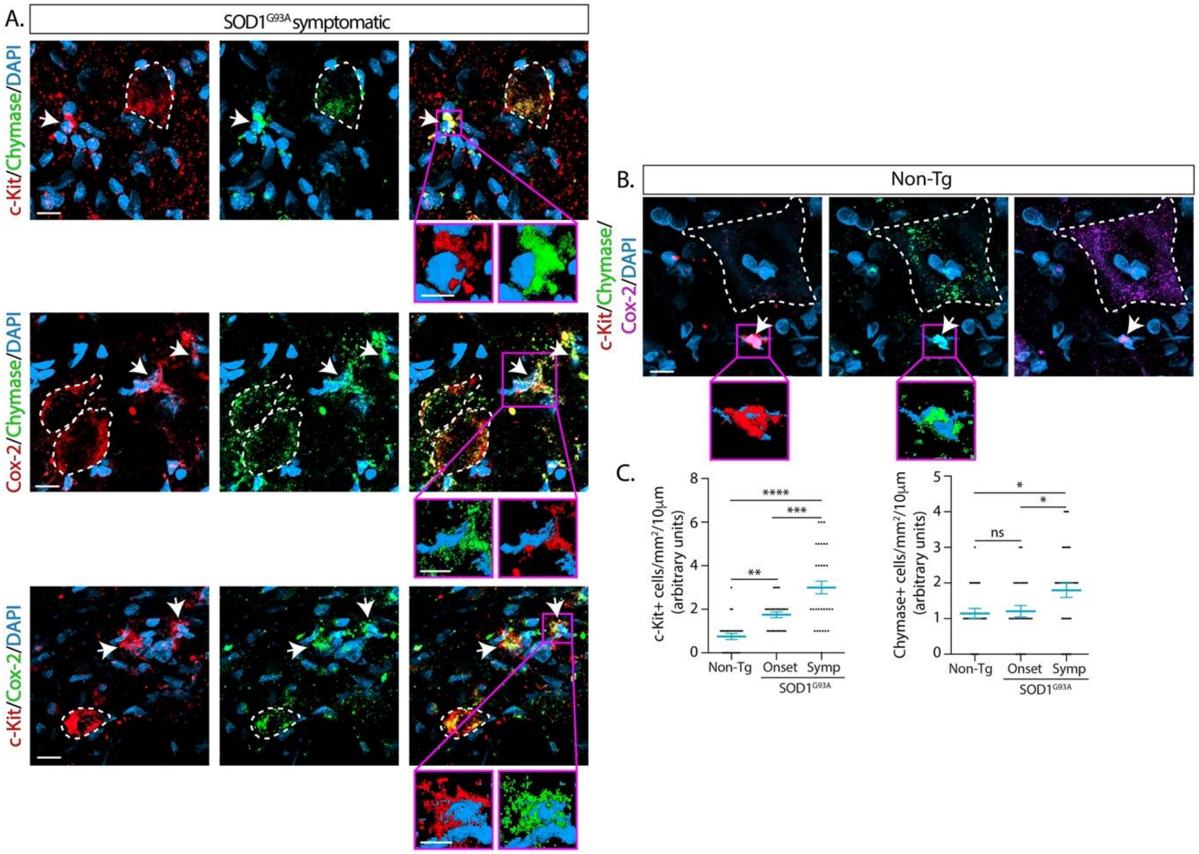

Fig. 3. Mast cells in the SOD1^{G93A} rat ventral horn. (A) Representative confocal images showing c-Kit+/chymase+ (left upper panels), Cox-2+/chymase+ (left middle panels) and c-Kit+/Cox-2+ (left bottom panels) MCs associated with motor neurons (white dotted lines) in the ventral horn of symptomatic SOD1^{G93A} lumbar spinal cord. Arrows indicate typical mast cells bearing granular morphology. Magenta squares indicate the area taken for 3D reconstructions of mast cells. (**B)** Representative confocal images of c-Kit+, chymase + and Cox-2 + MCs in Non-Tg rats. Arrows indicate small MCs. (**C)** Quantitative analysis of c-Kit+ (left) and chymase+ (right) MCs in the ventral horn of the lumbar spinal cord among conditions. Quantitative data are expressed as mean ± s.e.m. Data were analyzed by Kruskal-Wallis followed by Dunn´s multiple comparison test with **p*<0,05, **p<0,01, ****p*<0,001 and ****p>0,0001 considered significant. *n* = 4 animals/condition. Scale bars: 10 μm.

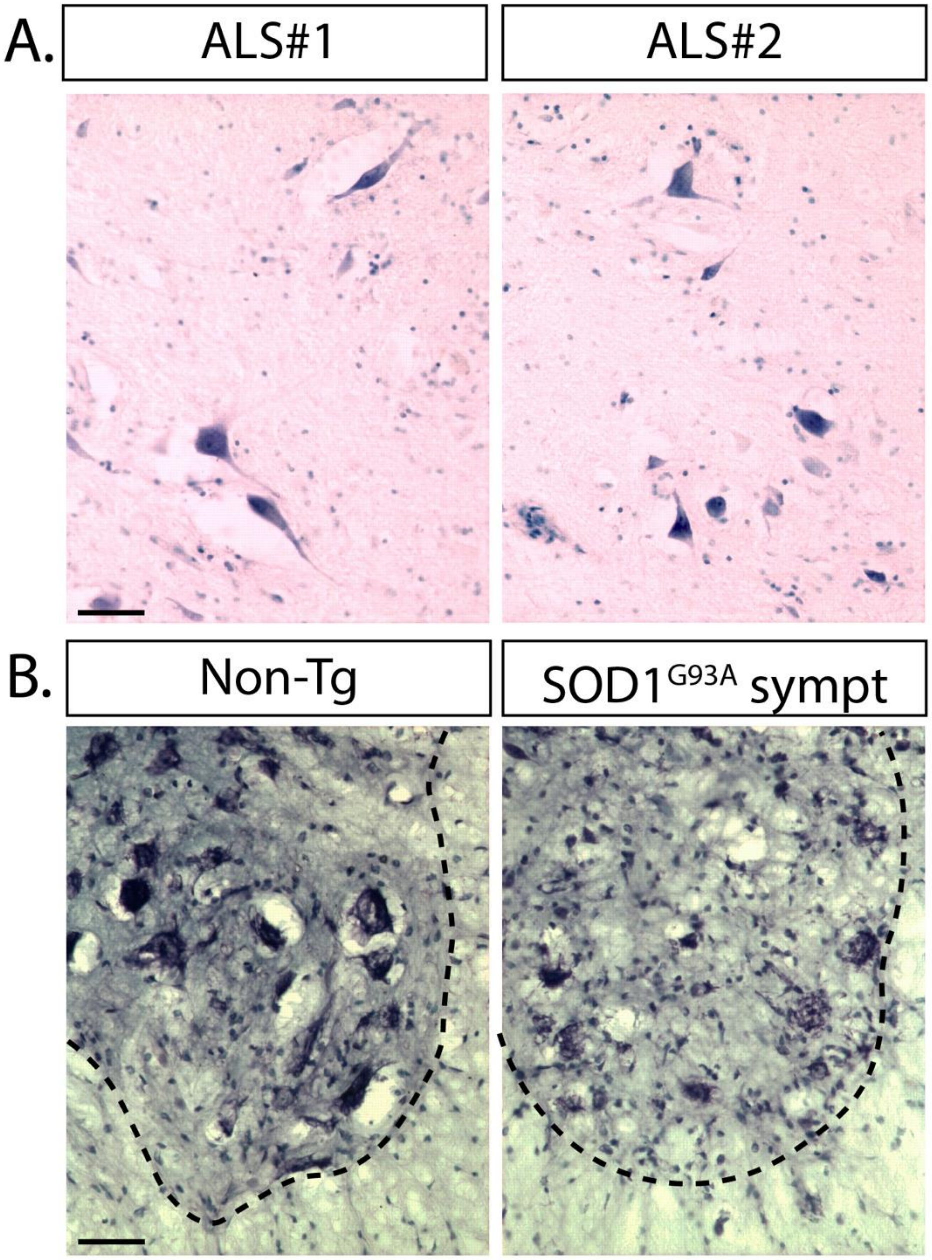

Fig. 4. Lack of metachromasia following toluidine blue staining in the spinal cord of ALS patients and mice. Representative histological images showing the lack of metachromatic MCs in sections stained with toluidine blue from **(A)** ALS patients and control donors, and **(B)** Non-tg and $SOD1^{G93A}$ symptomatic mice. Scale bars: 50 μm in (A) and 40 μm in (B).

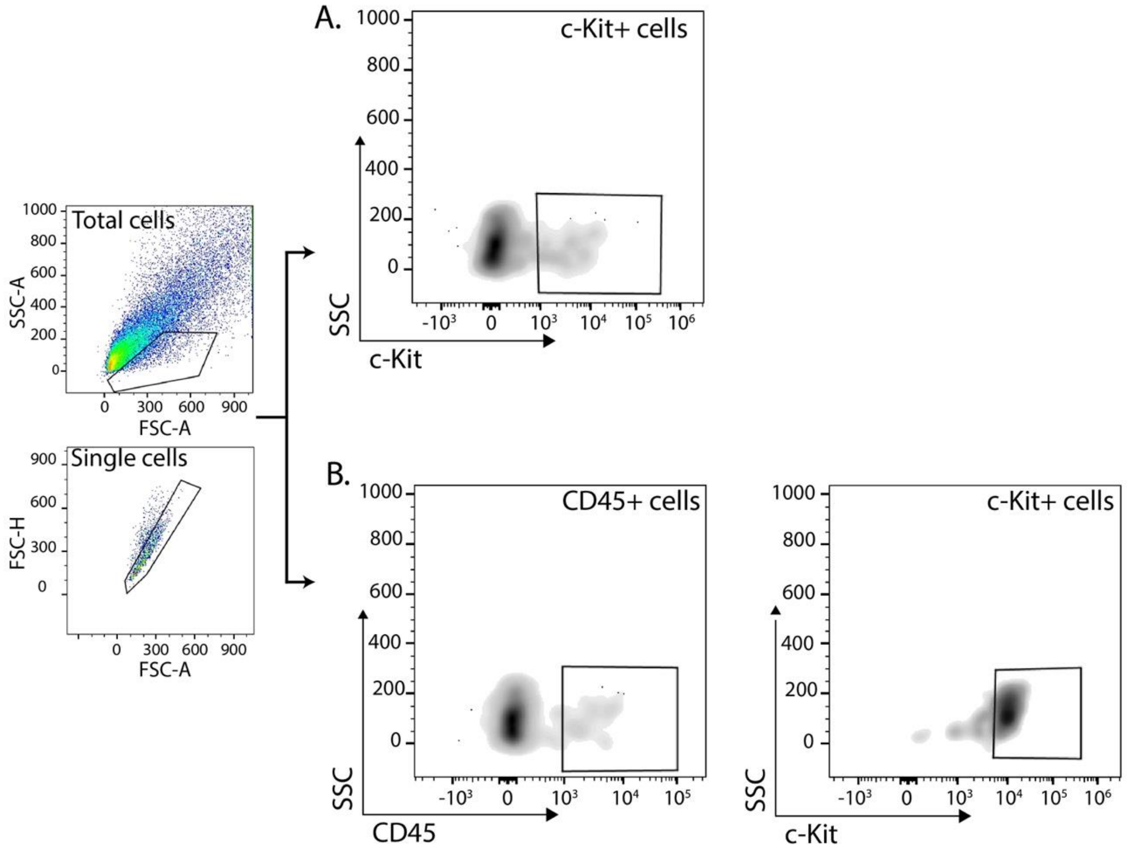

Fig. 5. Gating strategy in flow cytometry analysis of c-kit+ and CD45+ population in the spinal cord of ALS mice. Hematopoietic cells were labeled with primary antibodies against CD45 and c-Kit in a cell suspension obtained from the spinal of Non-Tg and SOD1^{G93A} symptomatic mice (150d). Mast cell population was analyzed following two different strategies. **(A)** c-Kit+ cell population was directly analyzed from total hematopoietic cell population, and **(B)** c-Kit+ cell population was selected among CD45+ cell population.

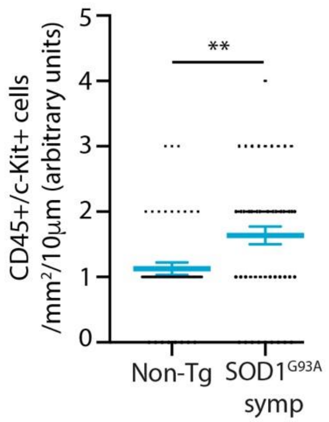

Fig. 6. Quantitative analysis of CD45+/c-Kit+ mast cells. The graph shows the quantitative analysis of CD45+/c-Kit+ in the surrounding of motor neurons in the ventral horn of the spinal cord of $SOD1^{G93A}$ mice compared to Non-Tg littermates. Quantitative data are expressed as mean ± s.e.m. Data were analyzed by Mann- Whitney test with ** p<0,001 considered significant. n=4 animals/condition.

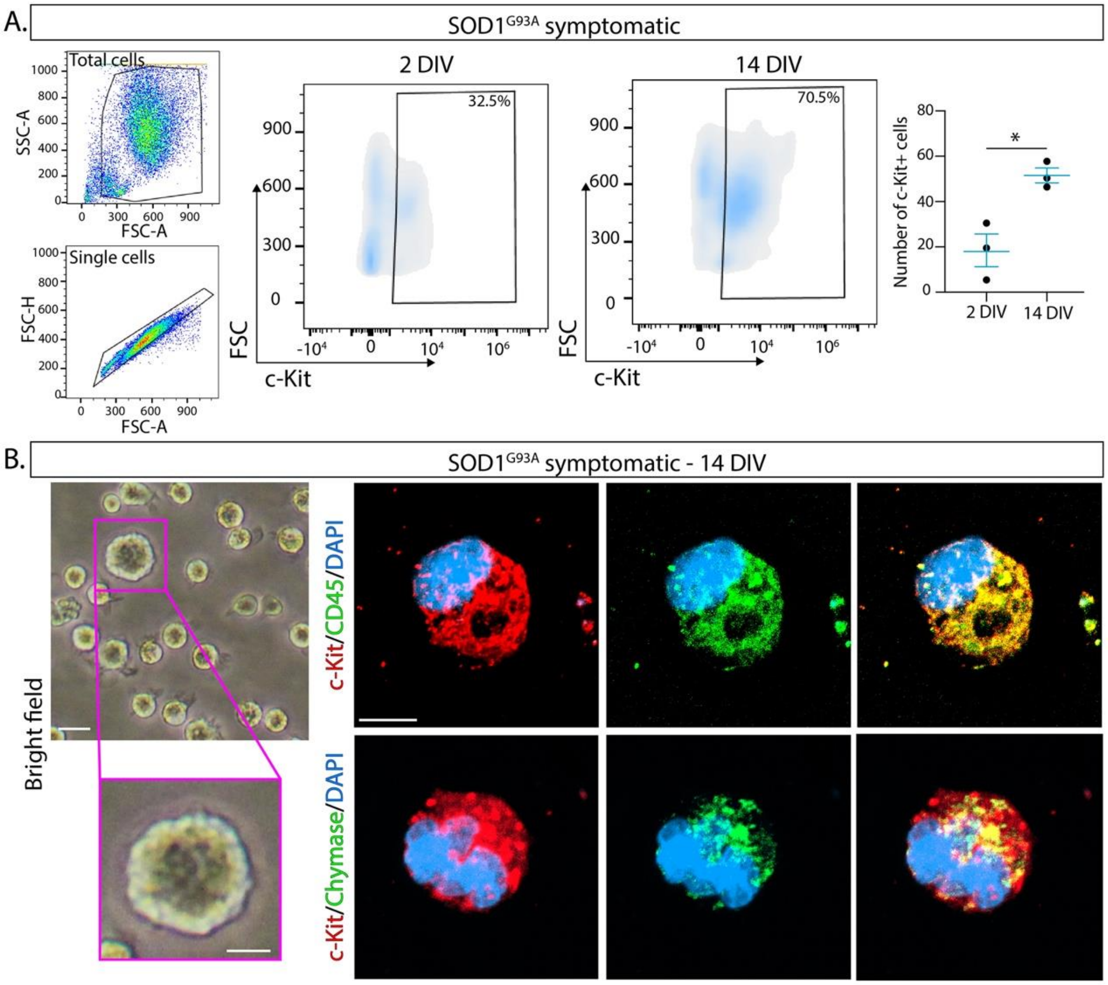

Fig. 7. Generation of c-Kit+ mast cells from the bone marrow of symptomatic SOD1^{G93A} rat. Bone marrows were cultured with IL-3 and SCF to induce MC differentiation, and c-Kit+ cell population was analyzed after 2 and 14 days by flow cytometry. (**A)** Representative plots showing gating strategy followed (left panels) and the c-Kit+ cell population analyzed at 2 and 14 days (right panels). The graph to the right shows the increase of c-Kit+ cell population from 2 to 14 days *in vitro*. (**B)** Representative cytological analysis of MCs isolated from SOD1^{G93A} bone marrow. Left panels show representative bright-field images of MCs. Magenta square shows the area taken for high magnification analysis. Right panels show immunocytochemical phenotyping of MCs after 14 days in culture assessed by confocal microscopy using staining for c-Kit (red), CD45 (green) and chymase (green). Quantitative data are expressed as mean ± s.e.m. Data were analyzed by Mann- Whitney test with *p<0,05 considered significant. n=3 animals/condition. Scale bars: 20 μm in (B) lower magnification, 10 μm in high magnification, and 5 μm in immunocytochemical analysis.

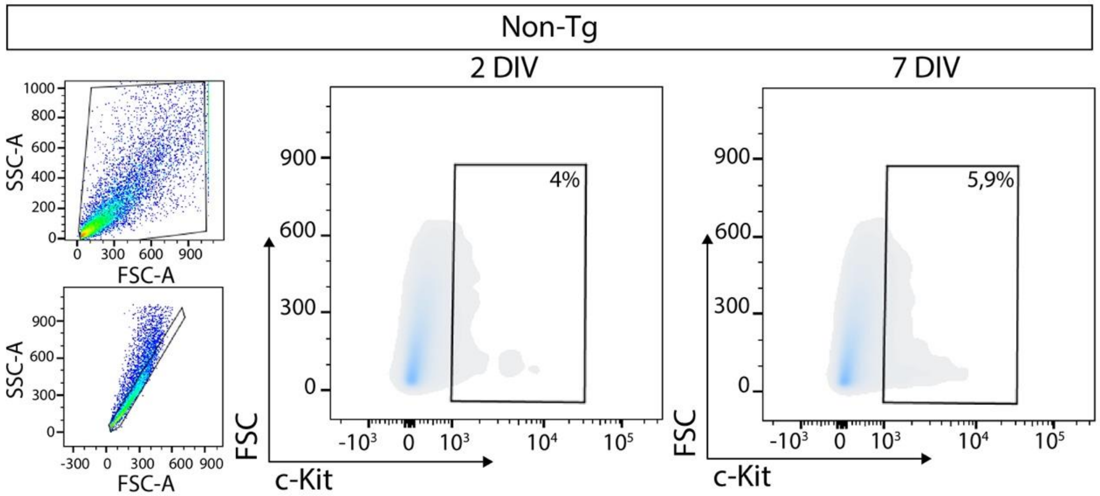

Fig. 8. Flow cytometry analysis of c-Kit+ mast cells isolated from the spinal cord of Non-Tg mice. Primary cultures of spinal cord of Non-Tg animals were maintained in presence IL-3 (20 ng/mL) and SCF (20 ng/mL) and c-Kit+ cell population was analyzed after 2 and 7 days *in vitro* by flow cytometry. Representative flow cytometry dot plots (left panels) showing the gating strategy followed and density plots (right panels) showing the expression of c-Kit at 2 and 7 days.

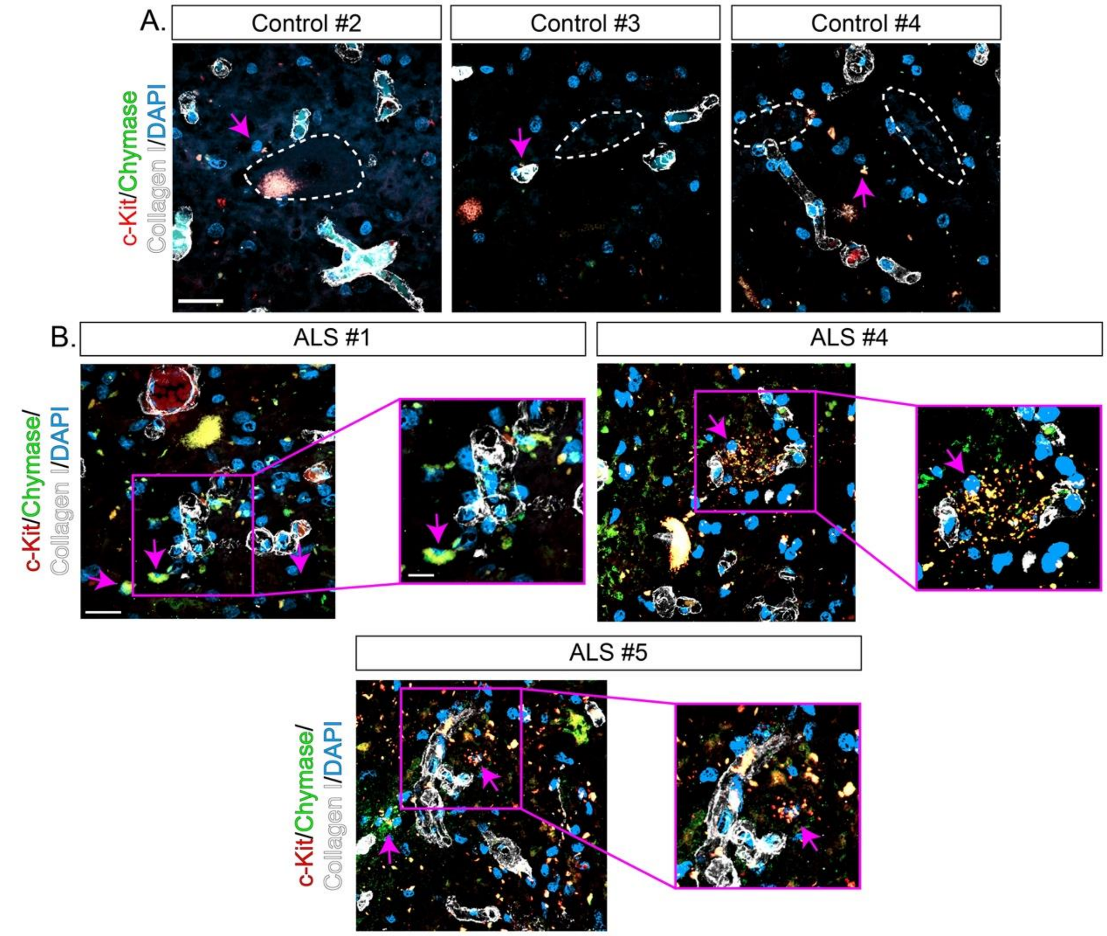

Fig. 9. Mast cells are associated with altered microvascular elements in ALS subjects but not in control donors. (A) Representative confocal microphotographs showing scarce number of c-Kit+/chymase+ MCs associated with microvascular elements stained with Collagen-I (white) in the ventral horn of the spinal cord of control donors. Magenta arrows indicate small MCs stained with both markers. (**B)** Representative confocal images of c-Kit+/chymase+ MCs (magenta arrows) associated with altered microvascular elements stained with Collagen-I (white) in the ventral horn of ALS subjects. Magenta squares show the area taken for high magnification analysis. Scale bars: 20 μm (low magnification panels) and 10 μm (insets).

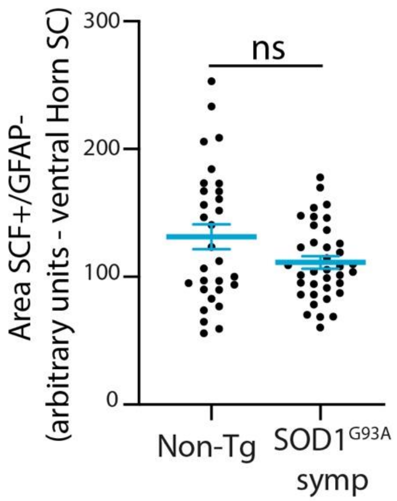

Fig. 10. Quantitative analysis of SCF immunoreactivity in motor neurons in the spinal cord of Non-Tg and symptomatic SOD1^{G93A} mice. Quantitative data are expressed as mean ± s.e.m. Data were analyzed by Mann- Whitney test. There was no significant difference among conditions. n=4 animals/condition.

**Subjet**	**Tissue**	**MRC grade**[a]	**EMGb denervation**	**Age (years)**	**Gender**	**Disease onset**	**Survival (Months)**	**Post-mortem tissue processing (hours)**	**Sporadic/ familial**
**ALS #1**	Lumbar Spinal Cord	3	Active and chronic	63	M	Leg	44	7.0	Sporadic
**ALS #2**	Lumbar Spinal Cord	1	Active	69	F	Leg	50	3.0	Sporadic
**ALS #3**	Lumbar Spinal Cord	3	Chronic	64	M	Leg	35	6.5	Sporadic
**ALS #4**	Lumbar Spinal Cord	3[c]	Active	59	F	Arm	26	13.0	Sporadic
**ALS #5**	Lumbar Spinal Cord	3	Active and chronic	75	M	Bulbar	55	4.3	Sporadic
Control #1	Lumbar Spinal Cord	-	-	68	M	-	-	19	-
Control #2	Lumbar Spinal Cord	-	-	59	M	-	-	9.5	-
Control #3	Lumbar Spinal Cord	-	-	90	M	-	-	4.5	-
Control #4	Lumbar Spinal Cord	-	-	62	M	-	-	3.0	-

Table 1. Clinical characteristics of ALS and control subjects included in the study. [a]Medical Research Council (MRC), muscle power grade at clinic visit prior to death. [b]EMG performed at time of ALS diagnosis. [c]Muscle showed predominant spasticity.
